# Microplastic exposure is associated with enhanced PI3K-Akt signaling in DSS-induced colitis: integrated bioinformatics analyses and *in vivo* validation

**DOI:** 10.3389/fphar.2026.1875549

**Published:** 2026-09-18

**Authors:** Lisha Lu, Yuanming Yang, Shunyong He, Shaogang Huang, Yulong Li

**Affiliations:** 1 The Affiliated People’s Hospital of Fujian University of Traditional Chinese Medicine, Fuzhou, China; 2 The First Affiliated Hospital of Guangzhou University of Chinese Medicine, Guangdong Clinical Research Academy of Chinese Medicine, Guangzhou, China; 3 The First Clinical Medical School of Guangzhou University of Chinese Medicine, Guangzhou, Guangdong, China

**Keywords:** bioinformatics, GMFA network, microplastics, molecular docking, toxicology, ulcerative colitis

## Abstract

**Background:**

Microplastics (MPs) are widely distributed in the environment and may aggravate ulcerative colitis (UC), but the underlying molecular mechanisms remain unclear.

**Methods:**

We integrated network toxicology, RNA-seq-based differential expression analysis, molecular docking, GeneMANIA-based functional association analysis, and experimental validation in a murine DSS-induced colitis model.

**Results:**

We identified 421 MP-related and 6,452 UC-related targets, including 265 overlapping targets. PIK3CD, PIK3CA, SRC, PTPN11, and EGFR were prioritized as hub targets with stable ligand binding. Enrichment analyses implicated the PI3K-Akt, JAK-STAT, T-cell receptor, and HIF-1 signaling pathways. In vivo experiments showed that MP exposure increased the colonic abundance of these five proteins and enhanced p-PI3K and p-AKT signals in DSS-induced colitis.

**Conclusion:**

MP exposure is associated with enhanced PI3K-Akt signaling in experimental colitis. These findings provide preliminary biological support for the PI3K-Akt pathway as a potentially relevant mechanism linking MP exposure to UC aggravation.

## 1 Introduction

Ulcerative colitis (UC) is a persistent and recurrent inflammatory bowel disease (IBD) characterized by mucosal inflammation, with the manifestation of abdominal pain, tenesmus, and bloody diarrhea (Le Berre et al., 2023). The incidence of UC is increasing, coinciding with the Industrial Revolution and a major ecological shift towards the modern environment (Estevinho et al., 2024). This epidemic pattern is especially evident in developing and recently developed countries in Asia and Africa. Research has predicted that the prevalence in East Asia is expected to increase 1.5 times, and in high-income Asia-Pacific and Southeast Asia, it is expected to increase 1.6 times by 2035 compared to 2020 (Olfatifar et al., 2021). Globally, the estimated number of IBD cases increased from 3.3 million in 1990 to 4.9 million in 2019, a rise of 47.5% (Wang et al., 2023), accounting for over $25 billion in annual direct healthcare costs in the United States (Singh et al., 2022). Therefore, UC is a global public health concern.

Microplastics (MPs), defined as plastic particles smaller than 5 mm, originate from the degradation and weathering of various plastics. Global analysis has revealed that the accumulated plastic waste in landfills or the natural environment amounted to approximately 4.9 billion metric tons between 1950 and 2015 and up to roughly 12 billion tons by 2050, inevitably leading to widespread plastic pollution (Geyer et al., 2017). MPs are widely distributed in ecosystems and human food systems and have been detected in various foods, condiments, drinking water, beverages, and atmospheric fallout (Huang et al., 2021). Human exposure to MPs in daily life through diet, drinking, and inhalation is inevitable (Huang et al., 2021). This exposure causes accumulation of MPs in various human tissues and biological samples, including the liver, lungs, placenta, kidneys, spleen, blood, sperm, and feces (Kozlov, 2024). Accumulated MPs can lead to several harmful effects on human organ systems, such as oxidative stress, inflammation, changes in immune function and energy use, reduced cell growth, damage to tissues, problems with organ development and function, changes in biochemical markers, and potential genetic and cancer risks (Ali et al., 2024), which raises concerns about their impact on public health.

The intestine is considered to be the primary line of defense against orally ingested MPs. Once ingested, they may impair intestinal barrier structures, alter intestinal flora, and induce mild pro-inflammatory responses and oxidative stress in healthy individuals (Ravindra et al., 2025). The integrity of the intestinal barrier and balanced intestinal microecology are closely associated with the development of UC. Accumulating evidence has shown that the disease procession in UC is correlated with MPs (Estevinho et al., 2024; Yan et al., 2022). The intestinal microenvironment of patients with UC is different from that of healthy individuals, and MPs can significantly increase intestinal injury and exacerbate intestinal pathology development. Research has also demonstrated that MP can affect macrophage function and promote local inflammation (Garcia et al., 2024). However, none of these studies has systematically provided a response of how MPs influence the UC procession. To address this public concern, the current study will investigate how MPs affect UC at a molecular level and how they interact, using network toxicology and molecular docking methods. By using these advanced methods, this study aimed to reveal how regular exposure to MPs could affect the initiation and development of UC and offer basic guidelines for future research on safer, non-toxic MPs. In line with transparency in the reporting of Artificial Intelligence (TITAN) guidelines, we have ensured that our use of AI in this study is transparently reported (Agha et al., 2025).

## 2 Methods

### 2.1 Toxicity analysis of MPs

A multicenter study from China identified eight major microplastics in urine and semen samples (Zhang et al., 2024). Therefore, we investigated eight types of microplastics: polystyrene (PS), polypropylene (PP), polycarbonate (PC), polyethylene (PE), polyvinyl chloride (PVC), polytetrafluoroethylene (PTFE), polyethylene terephthalate (PET), and acrylonitrile butadiene styrene (ABS). The SMILES and molecular formulae of MPs were acquired from PubChem (https://pubchem.ncbi.nlm.nih.gov/, accessed on 22 October 2024). ProTox 3.0 (https://tox.charite.de/, accessed on 22 October 2024), which integrates molecular similarity and machine-learning models, was employed to forecast oral toxicity and toxicity endpoints, while SwissADME (http://www.swissadme.ch/, accessed on 22 October 2024) was utilized to estimate gastrointestinal absorption.

### 2.2 Targets collection of MPs

Potential targets for MPs were identified and gathered from online databases, including Super-PRED (https://prediction.charite.de/, accessed on 22 October 2024), Swiss Target Prediction (http://www.swisstargetprediction.ch/, accessed on 22 October 2024), and ChEMBL (https://www.ebi.ac.uk/chembl/, accessed on 22 October 2024). The candidate targets from open sources were merged and deduplicated following conventional gene symbol transformation, with checking and human setting in the UniProt database.

### 2.3 Acquisition of UC related targets

To identify disease targets, two strategies were employed to obtain the targets of UC. The first strategy involved searching the public databases GeneCards (https://www.genecards.org, accessed on 22 October 2024) and NCBI gene (https://www.ncbi.nlm.nih.gov, accessed on 22 October 2024), which were searched with the keyword “ulcerative colitis”. The second strategy involved performing differential expression analysis on two gene expression profiles derived from an intestinal mucosal biopsy sample acquired from the Gene Expression Omnibus (GEO, https://www.ncbi.nlm.nih.gov/geo/, accessed on 22 October 2024). Differentially expressed genes (DEGs) were identified by the limma package (version 3.58.1) with the screening criteria of adjust p value <0.05 and |log2 fold change (FC)| > 1. Subsequently, disease targets were identified by merging the search results from public databases with genes with differential expression from GEO datasets. A Venn diagram was utilized to identify the shared targets of MPs and UC, which may serve as prospective targets for the toxicity of MPs in UC.

### 2.4 Analysis of function process and molecular pathways

Enrichment analysis of Gene Ontology (GO) and Kyoto Encyclopedia of Genes and Genomes (KEGG) was conducted to elucidate the functional processes and molecular pathways associated with the common target of MPs-induced toxicity in ulcerative colitis, utilizing the R package ClusterProfiler (version 4.2.2) and org.Hs.eg.DB (version 3.14.0). The species was restricted to “*Homo sapiens*,” and any term with an adjusted p-value below 0.05 was considered significantly enriched. The GO keywords and KEGG pathways were depicted in the results ranked by generation and plotted using the ggplot2 package (version 3.3.6). Given that GO enrichment might yield an extensive list of key phrases with considerable redundancy, the R package rrvgo (version 1.6.0) was utilized to simplify the enrichment results, which were subsequently visualized using a heatmap.

### 2.5 Construction of protein-protein interaction network and hub target analysis

The STRING database (https://string-db.org, accessed on 25 October 2024) was employed to construct the protein-protein interaction (PPI) network with a minimum required interaction score of 0.9 and a species restriction of *H. sapiens*. The TSV file for the interaction was downloaded and visualized by Cytoscape (version 3.9.0). To scientifically identify the hub gene inside the network, three distinct plugins (NetworkAnalyzer, MCODE, and cytoHubba) were employed for structural and core node analysis. The node with a degree equal to or exceeding the median, the cluster with the highest MCODE score, and the top 10 ranked nodes based on MCC in cytoHubba were identified as prospective key targets, with the final hub targets determined by integrating the results of the three distinct techniques.

### 2.6 Molecular docking of MP and hub targets

The binding affinity between the components of the MPs and core targets was investigated by molecular docking techniques. The SDF structure file of the compounds was downloaded from PubChem (https://pubchem.ncbi.nlm.nih.gov, accessed on 30 October 2024) and subsequently converted to a PDB file with OpenBabel (version 3.1.1). Then, AutoDock (version 1.5.7) was employed to eliminate water, add hydrogen, and save the file in PDBQT format. The crystal structure of the hub target was retrieved from the Protein Data Bank database (https://www.rcsb.org, accessed on 30 October 2024). PyMOL (version 3.0.3) was utilized to remove water molecules and ligands from the protein and save it in PDB format. AutoDock was employed to obtain the PDBQT protein format. AutoDock Vina was employed for molecular docking and binding energy calculations, and the results were shown using PyMOL and Discovery Studio Visualizer. We selected a binding energy of approximately −5.0 kcal/mol as the criterion for stable binding in our research.

### 2.7 GeneMANIA-based functional association network analysis

A novel approach within the GeneMANIA framework was employed to comprehensively evaluate genes functionally associated with hub targets that exhibited strong binding energy. GeneMANIA-based functional association (GMFA) network analysis attempts to identify 10 more genes for every hub gene, focusing on the genes that have the strongest connections within the gene–gene network. This analysis focused on three essential parameters: co-expression, genetic interaction, and physical interaction, thereby markedly improving the accuracy of therapeutic target identification. By comprehensively considering these factors, we have attained a more nuanced understanding of gene function, enabling the identification of robust toxicity targets with complex roles in disease mechanisms. Subsequently, we integrated all newly identified genes with hub targets exhibiting superior binding energy, establishing a GMFA-based Expanded Database (GMFA-ED) for MPs-induced UC. The integration results provide a more comprehensive foundation for identifying potential toxicity targets in MPs-induced UC. GO and KEGG pathway enrichment analyses were conducted for the genes in GMFA-ED. Finally, a component-target-pathway network was constructed to elucidate the intricate interactions in MPs-induced UC.

### 2.8 Animal experiment and experimental validation design

To validate the candidate key pathway identified by the *in silico* analyses, an animal-based experimental validation was performed. Eighteen C57BL/6J mice were housed in an animal facility maintained at 20 °C ± 2 °C, subjected to a 12-h light and 12-h dark cycle, with humidity levels about 55%–65%. Experiments commenced following 1 week of acclimatization. The 18 mice were randomly divided into three groups (6 mice per group) based on their body weight using a random number table: normal control group, dextran sodium sulfate (DSS) group (colitis group), DSS plus MPs group (colitis + MPs group). DSS (MP Biomedals, United Kingdom) was solubilized in distilled water to create a 3% DSS solution. The stock concentration of MPs (Zhongkeleiming, Beijing) was 50 mg/mL. Before daily intragastric administration, the stock solution was diluted with ultrapure water to a working concentration of 5 mg/mL, followed by sufficient ultrasonic oscillation to achieve uniform dispersion of MPs. Mice in the colitis group and colitis plus MPs group were administered a 3% DSS solution for a duration of 7 days. Subsequently mice in the colitis plus MPs group were treated with microplastic (5 mg/kg/d, suspended in 100 µL of sterile deionized water) by gavage for seven consecutive days. Mice in the normal control group and the colitis group were simultaneously given the same volume of sterile deionized water by gavage as the control. From day 1 onwards, body weight variations, stool consistency and rectal bleeding of mice from all three groups were recorded daily, and the Disease Activity Index (DAI) was calculated accordingly. On day 15, the mice were sacrificed under anesthesia, the colon tissues were rapidly isolated and colon lengths were measured. Among them, the tissues used for HE staining were fixed and preserved in 4% paraformaldehyde, while the tissues used for immunofluorescence, western blot detection and Enzyme-Linked Immunosorbent Assay were rapidly frozen in liquid nitrogen and then transferred to a −80 °C refrigerator for storage and future use. The morphology, particle-size distribution, and zeta potential of the MPs preparation are provided in Supplementary Figures S1-S3.

### 2.9 Reagents, antibodies, and consumables

Key reagents and consumables were obtained from Thermo Fisher Scientific, Abcam, Proteintech, Servicebio, Solarbio, Jiangsu MeiMian, and Beijing Zhongkeleiming, according to the supplied reagent records. ELISA kits for interleukin-6 (IL-6; Jiangsu MeiMian, MM-1011M2) and tumor necrosis factor-α (TNF-α; Jiangsu MeiMian, MM-0132M2) were used for cytokine quantification. For western blotting, the primary antibodies included AKT (rabbit, Proteintech, 10176-2-AP, 1:10,000), p-AKT (mouse, Proteintech, 66444-1-Ig, 1:8,000), PI3K (mouse, Proteintech, 60225-1-Ig, 1:40,000), p-PI3K (rabbit, Thermo Fisher Scientific, PA5-17387, 1:800), and β-actin (mouse, Proteintech, 66009-1-Ig, 1:10,000). For validation of the five predicted hub targets, the following primary antibodies were additionally used: PIK3CA (Mouse, Proteintech, 67071-1-Ig, 1:2000), PIK3CD (Rabbit, Proteintech, 21708-1-AP, 1:1,000), SRC (Rabbit, Proteintech, 11097-1-AP, 1:1,000), PTPN11 (Rabbit, Proteintech, 82503-1-RR, 1:20,000), and EGFR (Rabbit, Proteintech, 18986-1-AP, 1:2000). The secondary antibodies were HRP-goat anti-rabbit IgG (Proteintech, RGAR001, 1:10,000) and HRP-goat anti-mouse IgG (Proteintech, RGAM001, 1:10,000). PVDF membranes, BCA protein assay kit, RIPA lysis and extraction buffer, 20× TBST buffer, antibody diluent, blocking buffer, goat serum, and ECL substrate were used according to the manufacturers’ instructions.

### 2.10 Western blotting, immunofluorescence staining and enzyme-linked immunosorbent assay

To evaluate PI3K-Akt pathway activation in MPs-exposed ulcerative colitis (UC) mice, immunofluorescence staining was performed for p-AKT and p-PI3K in colonic sections. Three biologically independent animals per group were analyzed (n = 3). DAPI was imaged in blue and p-AKT or p-PI3K in red using identical acquisition settings across groups. Positive area, integrated optical density (IOD), and measured area were quantified using Image-Pro Plus, and normalized density was calculated as IOD divided by measured area. Representative p-AKT and p-PI3K staining patterns are shown in Figures 1A,C, and the corresponding normalized-density quantification is shown in Figures 1B,D. Western blotting was performed to quantify PI3K, p-PI3K, AKT, and p-AKT protein abundance. Colonic PIK3CA, PIK3CD, SRC, PTPN11, and EGFR protein abundance was also assessed by western blotting. Representative blot images, cropped bands, and quantitative analyses are provided in Supplementary Figures S4-S7. The remaining immunofluorescence biological replicates are provided in Supplementary Figure S8, and the underlying quantification values are reported in Supplementary Tables S1 and S2. Colon-tissue concentrations of IL-6 and TNF-alpha were measured using the indicated ELISA kits according to the manufacturers’ instructions.

**FIGURE 1:**
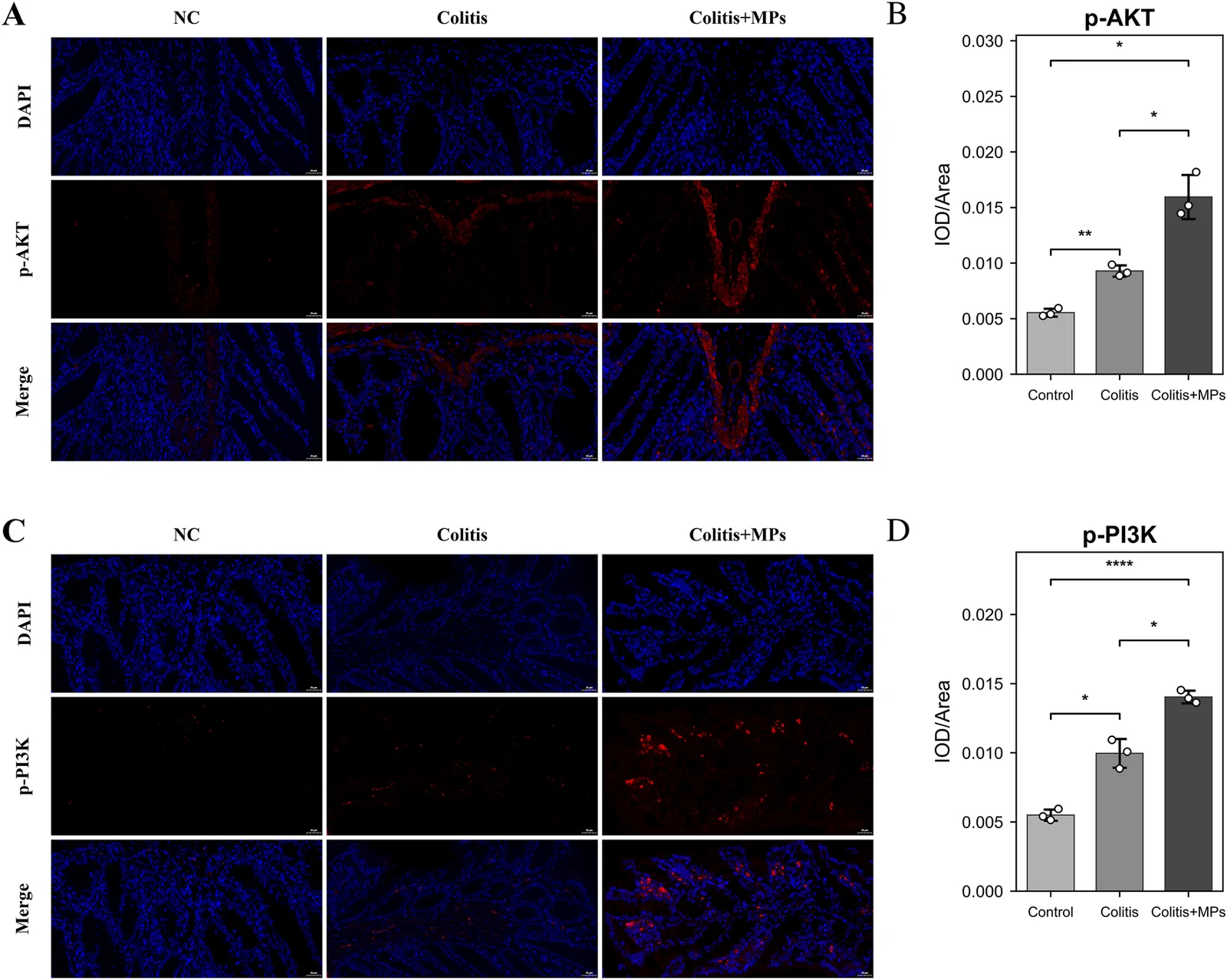
Immunofluorescence analysis of PI3K-Akt pathway activation. **(A)** Representative DAPI (blue), p-AKT (red), and merged images showing the regional distribution of p-AKT signal in the colonic mucosa. The apparent crypt-associated pattern is a morphology-based observation and does not establish epithelial-cell-specific localization; **(B)** normalized p-AKT fluorescence density, calculated as integrated optical density divided by measured area; **(C)** representative DAPI (blue), p-PI3K (red), and merged images; and **(D)** normalized p-PI3K fluorescence density. Individual biological replicate values are overlaid on the bars in **(B)** and **(D)**. Data are presented as mean ± SD from three biologically independent animals per group (n = 3) and were analyzed using Welch’s ANOVA followed by Games-Howell multiple-comparisons tests. *P < 0.05; **P < 0.01; ***P < 0.001; Scale bars = 25 μm. Additional biological replicates are shown in Supplementary Figure S8, and the underlying measurements are provided in Supplementary Tables S1 and S2. NC, normal control.

### 2.11 Statistical analysis of experimental validation

All statistical analyses were performed using SPSS version 25.0 (IBM, Armonk, NY, United States). Continuous measurement data were expressed as mean ± standard deviation (mean ± SD). The Shapiro–Wilk test and Levene’s test were separately used to assess data normality and variance homogeneity, respectively. Intergroup comparisons were chosen according to data distribution characteristics: one-way ANOVA with Tukey’s HSD *post hoc* tests for normally distributed, homoscedastic data; Welch’s ANOVA followed by Games–Howell tests for normal data with unequal variances; and Kruskal–Wallis H test paired with Dunn’s test with Bonferroni correction for non-normal datasets. All statistical tests were two-sided, and P < 0.05 was defined as the threshold of statistical significance.

## 3 Results

### 3.1 Toxicological study of MPs

The chemical details of the eight microplastics are presented in Table 1, including their molecular formulas, SMILES structures, toxicity prediction results, and gastrointestinal absorptions. The lethal dosage prediction results from ProTox 3.0 categorized the various components of MPs into their respective toxicity classes; the lowest class number indicates the highest oral toxicity. PTFE is categorized as class three, whereas PS, PVC, and ABS are classified as class four. PC and PET were classified as class five, while PP and PE were designated as class six. Regarding organ toxicity, PS, PE, PVC, and ABS show neurotoxicity, whereas PP and PET exhibit nephrotoxicity. In terms of toxicity endpoints, all eight microplastics could compromise the blood-brain barrier, whereas PS, PVC, PTFE, and ABS possessed carcinogenic potential. Except for PP and PET, the other types of MPs exhibit ecotoxicity. The toxicity radar chart shown in Figure 2 aims to swiftly display the certainty of positive toxicity outcomes in relation to the class average. SwissADME also analyzes the gastrointestinal absorption of MPs, indicating that PET and ABS have high absorption levels in the gastrointestinal tract, whereas others are low.

**TABLE 1:** Basic information of microplastics and prediction of toxicity of chemicals with ProTox 3.0.

Microplastics (MP)	SMILES	Molecular formula	LD50 (mg/kg)	Toxicity class	Organtoxicity	Toxicity end points	GI absorption
Polystyrene (PS)	C=CC1=CC=CC=C1	C8H8	316	4	Neurotoxicity	Carcinogenicity; BBB-barrier; ecotoxicity	Low
Polypropylene (PP)	CCCCCCCC[C@@H]1[C@@H](O1)CCCCCCCCCCCC(=O)O	C22H42O3	16,000	6	Nephrotoxicity	BBB-barrier	Low
Polycarbonate (PC)	CC(C)(C1=CC=C(C=C1)O)C2=CC=C(C=C2)O.CC(C)(C1=CC=C(C=C1)O)C2=CC=C(C=C2)O.C(=O)([O-])[O-]	C31H32O7-2	5,000	5	—	BBB-barrier; ecotoxicity	Low
Polyethylene (PE)	CCCCC=C.C=C	C8H16	5,050	6	Neurotoxicity	BBB-barrier; ecotoxicity	Low
Polyvinyl chloride (PVC)	C=CCl	C2H3Cl	500	4	Neurotoxicity	Carcinogenicity; BBB-barrier; ecotoxicity	Low
Polytetrafluoroethylene (PTFE)	C(=C(F)F)(F)F	C2F4	268	3	—	Carcinogenicity; BBB-barrier; ecotoxicity	Low
Polyethylene terephthalate (PET)	C1=CC(=CC=C1C(=O)O)C(=O)O.C(CO)O	C10H12O6	2,340	5	Nephrotoxicity	BBB-barrier	High
Acrylonitrile butadiene styrene (ABS)	C=CC=C.C=CC#N.C=CC1=CC=CC=C1	C15H17N	1,072	4	Neurotoxicity	Carcinogenicity; BBB-barrier; ecotoxicity	High

Toxicity class, 1: fatal if swallowed (LD50 ≤ 5); 2: may be fatal if swallowed (5 < LD50 ≤ 50); 3: toxic if swallowed (50 < LD50 ≤ 300); 4: harmful if swallowed (300 < LD50 ≤ 2000); 5: may be harmful if swallowed (2000 < LD50 ≤ 5,000); 6: non-toxic (LD50 > 5,000). LD, Lethal dose; GI, gastro-intestinal; BBB, blood–brain barrier.

**FIGURE 2:**
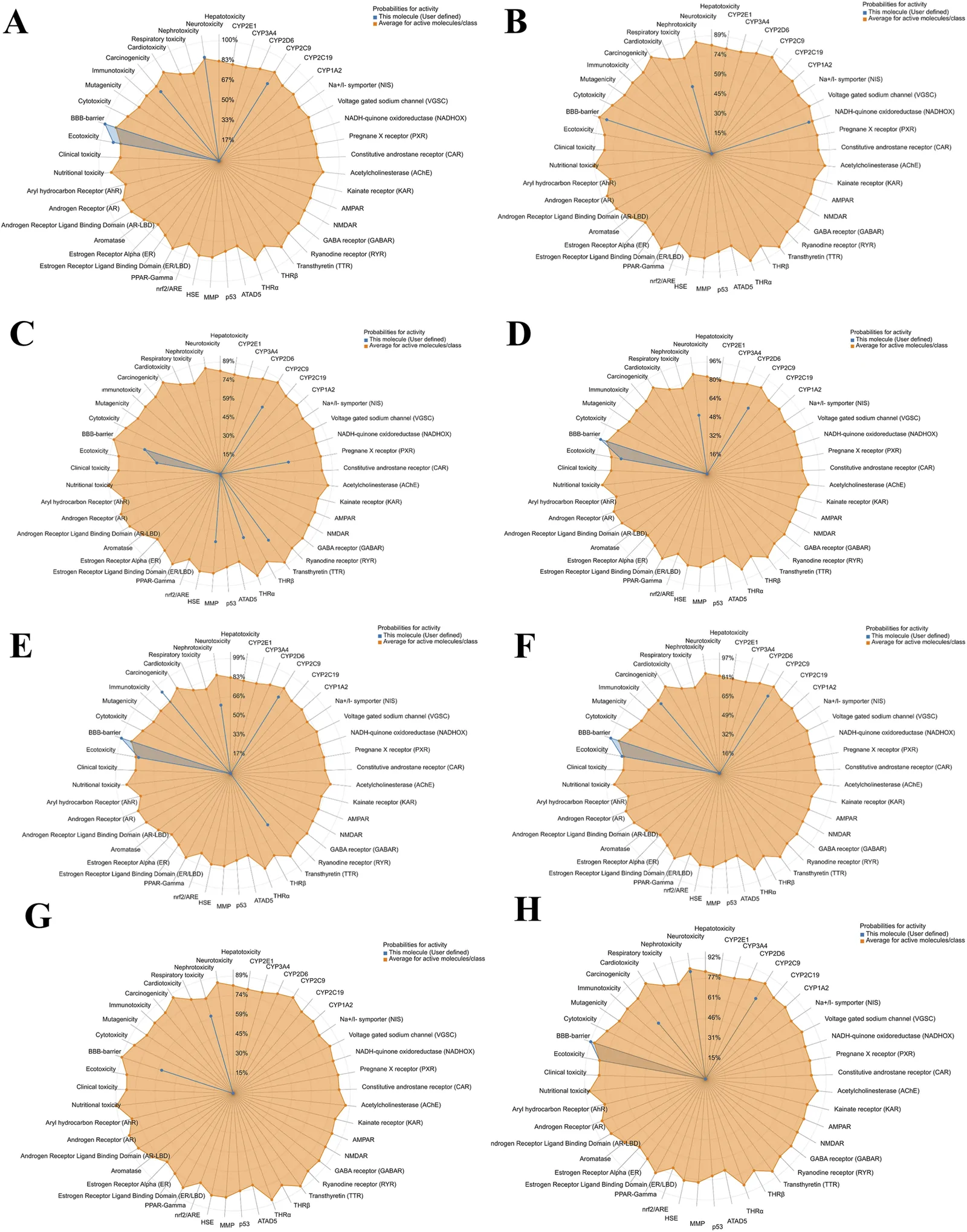
Prediction of toxicity of different chemicals with ProTox 3.0. **(A)** Polystyrene (PS); **(B)** Polypropylene (PP); **(C)** Polycarbonate (PC); **(D)** Polyethylene (PE); **(E)** Polyvinyl Chloride (PVC); **(F)** Polytetrafluoroethylene (PTFE); **(G)** Polyethylene Terephthalate (PET); **(H)** Acrylonitrile butadiene styrene (ABS).

### 3.2 Toxicity target of MPs inducing UC

After combining and deduplicating the data from open-source databases, we found 421 distinct targets for all eight microplastic components. The analysis revealed 77 targets with PS, 105 with PP, 74 with PC, 149 with PE, 167 with PVC, 105 with PTFE, 83 with PET, and 89 with ABS (Figure 3). Two open-source databases and two GEO datasets were used to gather disease targets. With the criteria of adjust p value <0.05 and |log2 fold change (FC)| > 1, GSE87466 identified 801 genes that were expressed differently, whereas GSE87473 identified 721 genes with similar differences. NCBI Gene obtained 909 targets, whereas GeneCards acquired 5,823 targets. After integration and deduplication, 6452 UC targets were obtained (Figure 3). By looking at both sets of results, we found 265 targets that are common to both MPs and UC; these shared targets are believed to be the ones that cause toxicity in MPs, leading to UC.

**FIGURE 3:**
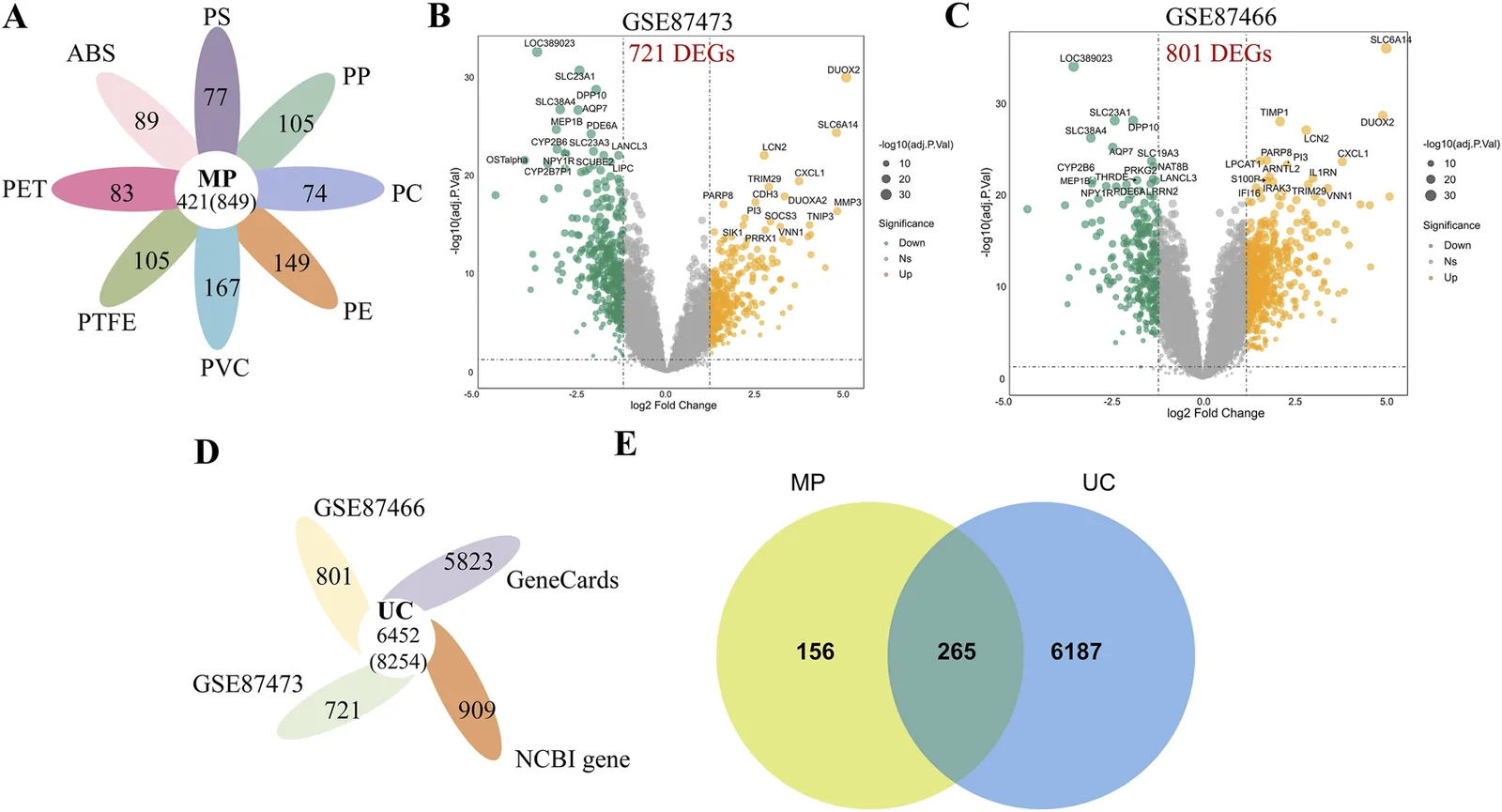
Targets of MPs and UC. **(A)** Number of target genes associated with the different MPs in open-source databases. **(B)** Differentially expressed genes in UC from GSE87473. **(C)** Differentially expressed genes in UC from GSE87466. **(D)** Number of UC-related genes obtained from open-source databases and GEO datasets. **(E)** Common targets between MPs and UC.

### 3.3 Enrichment of toxicity target

The R package ClusterProfiler was used to analyze the GO function and KEGG pathway of the toxicity target. A total of 754 GO terms were enriched, including 600 biological processes (BP), 51 cellular components (CC), and 103 molecular functions (MF), with an adjusted P value <0.05. The top 10 terms ranked by the gene ratio in each category are illustrated in Figure 4A. To avoid excessive overlap in the GO BP results, simpler findings were created using rrvgo and are shown in a heatmap (Figure 4B). The results indicate that the biological processes related to MPs toxicity mainly involve how cells respond to chemical stress, environmental changes, development of the digestive system, movement and growth of epithelial cells, and movement and sticking of white blood cells. The important components of the cell are the membrane raft, membrane microdomains, vesicle lumen, plasma membrane, focal adhesion, and endosome lumen. Molecular functions include tasks such as attaching to DNA transcription factors, working as protein tyrosine kinases, connecting with amides, interacting with phosphatases, binding to nuclear receptors, and functioning as ligand-activated transcription factors.

**FIGURE 4:**
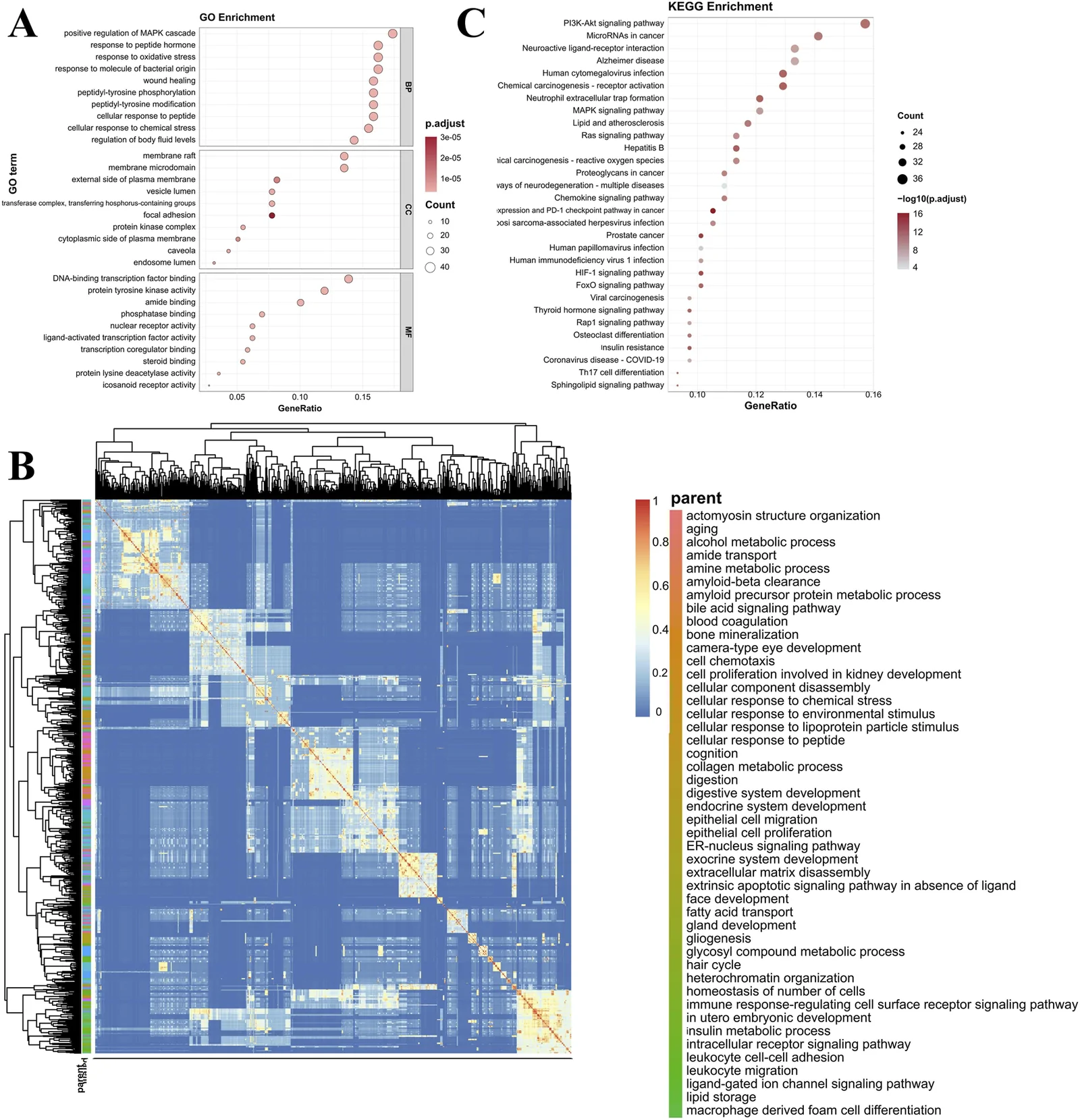
Enrichment analysis of common targets between MPs and UC. **(A)** Dot plot displaying the enriched GO biological process (BP), cellular components (CC) and molecular function (MF). **(B)** Clustering heatmap displaying the simplified results of GO BP. **(C)** Dot plot displaying the enriched KEGG pathway.

In the KEGG enrichment analysis, a total of 170 KEGG pathways associated with the toxicity targets were acquired, with an adjusted P value <0.05. The top 30 terms ranked by gene ratio are presented in Figure 4C. The KEGG pathways associated with MPs-induced UC mostly encompass PI3K-Akt signaling pathways, neutrophil extracellular trap formation, MAPK signaling processes, HIF-1 signaling pathways, Th17 cell differentiation, etc. All of these pathways are strongly associated with inflammation and immunological responses.

### 3.4 PPI network and hub targets

The PPI network was constructed by importing 265 common targets into the STRING database, with a minimum required interaction score of 0.9 and restricting the species to *H. sapiens* (Figure 5A). The TSV profile was downloaded and visualized using Cytoscape. NetworkAnalyzer was used to evaluate degree centrality, and the complete degree-ranked PPI network is provided in Supplementary Figure S9. MCODE was used to identify the most densely connected functional module (Figure 5B), whereas cytoHubba was used to identify the top 10 nodes ranked by maximal clique centrality (Figure 5C). Intersecting the results of the degree, MCODE, and cytoHubba analyses identified seven hub targets: SRC, PIK3R1, PIK3CA, PIK3CB, PIK3CD, PTPN11, and EGFR (Figure 5D).

**FIGURE 5:**
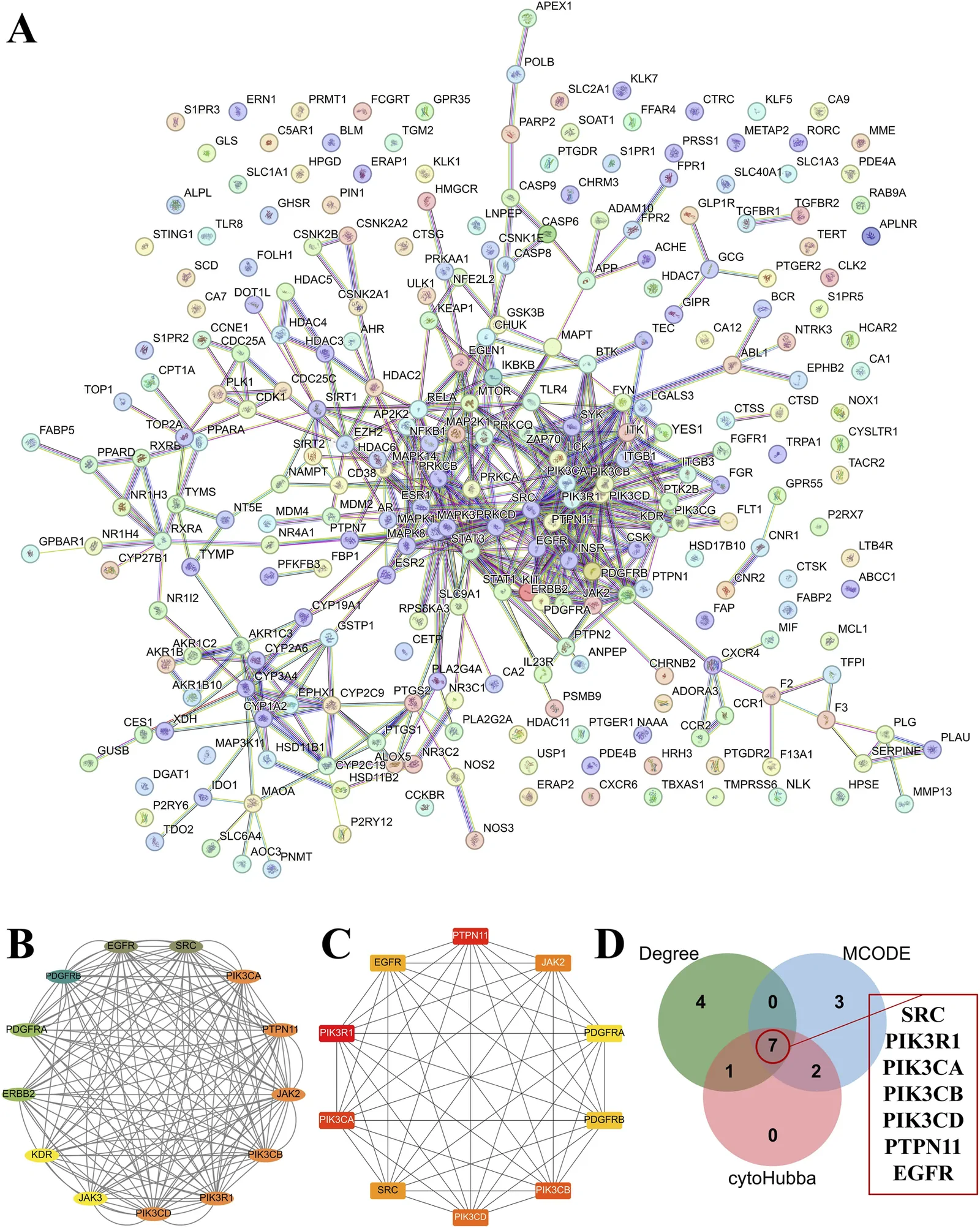
PPI network and hub-target identification. **(A)** PPI network constructed with a minimum required interaction score of 0.9. **(B)** Densely connected module with the highest MCODE score. **(C)** Top 10 nodes ranked by maximal clique centrality in cytoHubba. **(D)** Intersection of hub targets identified by degree, MCODE, and cytoHubba analyses. The complete degree-ranked PPI network is provided in Supplementary Figure S9.

### 3.5 Results of molecular docking

For molecular docking, we used hub targets found in the PPI. The corresponding MPs’ lists of hub targets were retrieved with target profiles, and we found that ABS did not target any hub targets. The 3D structure of the target protein, PIK3CB, was not retrieved from the PDB database. Consequently, ABS and PIK3CB were excluded from the molecular docking analysis. Generally, a binding energy below 0 kcal/mol indicates that ligands and targets can bind spontaneously, whereas a binding energy less than −5.0 kcal/mol indicates stable binding. Furthermore, a lower binding energy represents a stronger binding affinity and a higher probability of interaction. Our results indicate that all the MPs’ ligands can spontaneously bind to the core targets with the binding energy less than 0, and seven ligand-protein complexes (including four chemicals and five targets) exhibit stable binding with the binding energies less than −5.0 kcal/mol (Table 2). Notably, PP binding to EGFR has the lowest binding energy (−8.4 kcal/mol) and demonstrates the strongest binding affinity and stability. Therefore, we show pictures in 2D and 3D, along with the distances and hydrogen bonds of how the ligand and protein interact, using the PP and EGFR complex as an example (Figure 6).

**TABLE 2:** Molecular docking of hub targets and microplastics.

Molecular	Targets	Bind energy(kcal/mol)
PS	PIK3CA	−5.7
PP	PIK3R1	−4.6
	SRC	−6.4
	PIK3CD	−8.3
	PTPN11	−6.4
	EGFR	−8.4
PC	PIK3R1	−4.6
	PTPN11	−6.4
PE	PIK3CD	−4.1
	PIK3R1	−2.8
	EGFR	−4.2
PVC	PIK3CA	−2.4
	PTPN11	−2.2
	EGFR	−2.5
	SRC	−2.3
PTFE	PIK3R1	−3
PET	PIK3R1	−4.6
	PTPN11	−5.5

**FIGURE 6:**
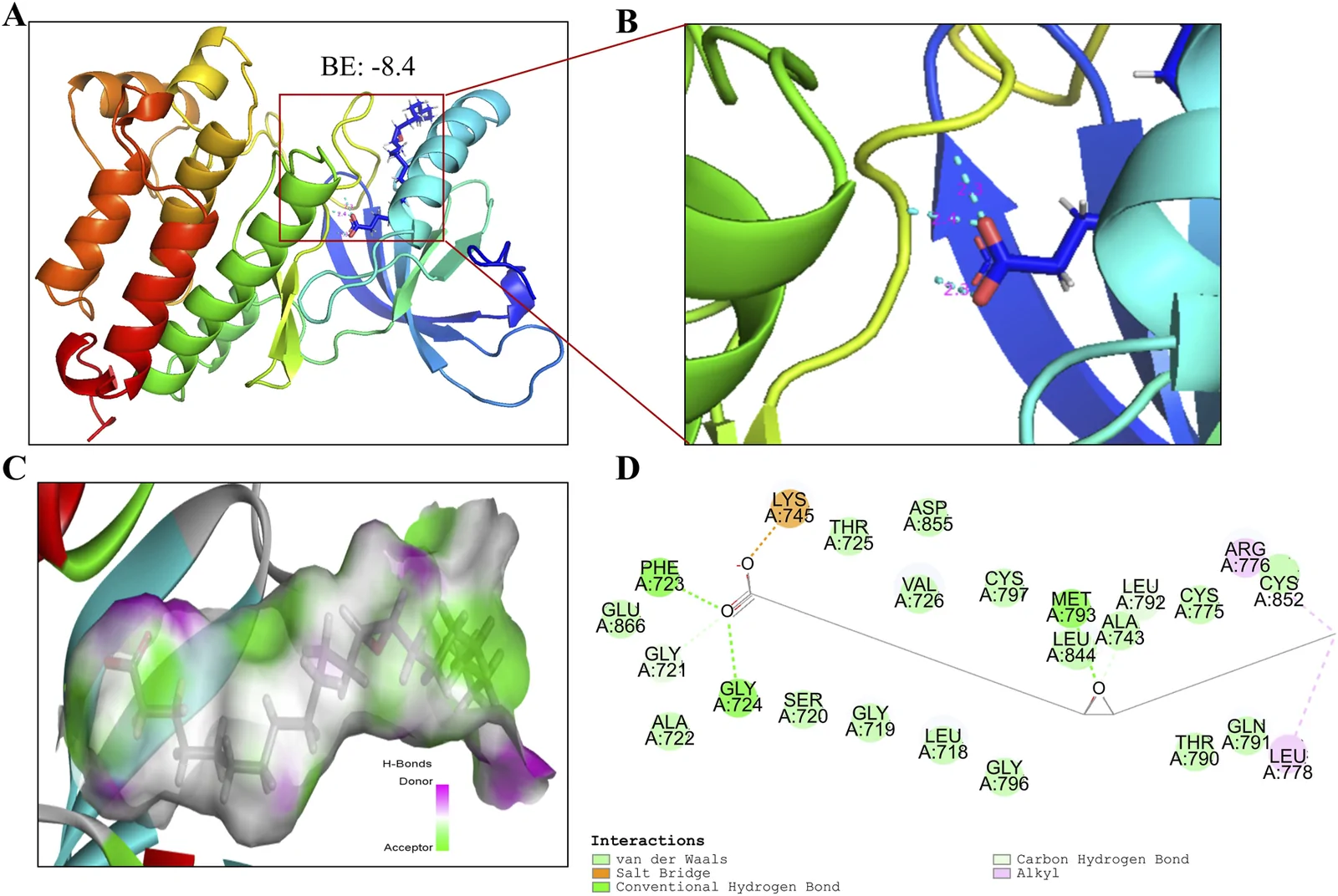
Molecular docking of PP and EGFR. **(A)** 3D view of ligand-protein interactions. **(B)** Distance of ligand-protein interactions. **(C)** Hydrogen bonds of ligand and protein. **(D)** 2D view of ligand-protein interactions.

### 3.6 GMFA network and enrichment analysis

Five targets (PIK3CD, PIK3CA, SRC, PTPN11, and EGFR) that exhibited stable ligand binding were utilized for GMFA network analysis. We expanded the gene list of these five targets by identifying 10 additional genes with the most robust associations within the gene-gene network for each hub target, resulting in GMFA-ED (Figure 7). After deduplication and integration, the GMFA-ED dataset comprised 51 unique targets and formed a functional gene network incorporating co-expression, co-localization, genetic interactions, pathways, physical interactions, shared protein domains, and predicted interactions (Supplementary Figure S10). GO and KEGG enrichment analyses were then performed to elucidate the biological functions and pathways represented in GMFA-ED. GO analysis indicated enrichment in biological processes relevant to UC development, including cellular responses to environmental stimuli, epithelial cell migration and proliferation, immune responses, regulation of phosphatidylinositol 3-kinase activity, and wound healing (Figure 8A). KEGG analysis highlighted pathways including PI3K-Akt, JAK-STAT, T cell receptor, and HIF-1 signaling (Figure 8B). The top 20 KEGG pathways ranked by gene ratio were used to construct the compounds-targets-pathways network. PTFE showed no stable binding to the hub targets and was therefore not displayed in the interaction network (Figure 8C).

**FIGURE 7:**
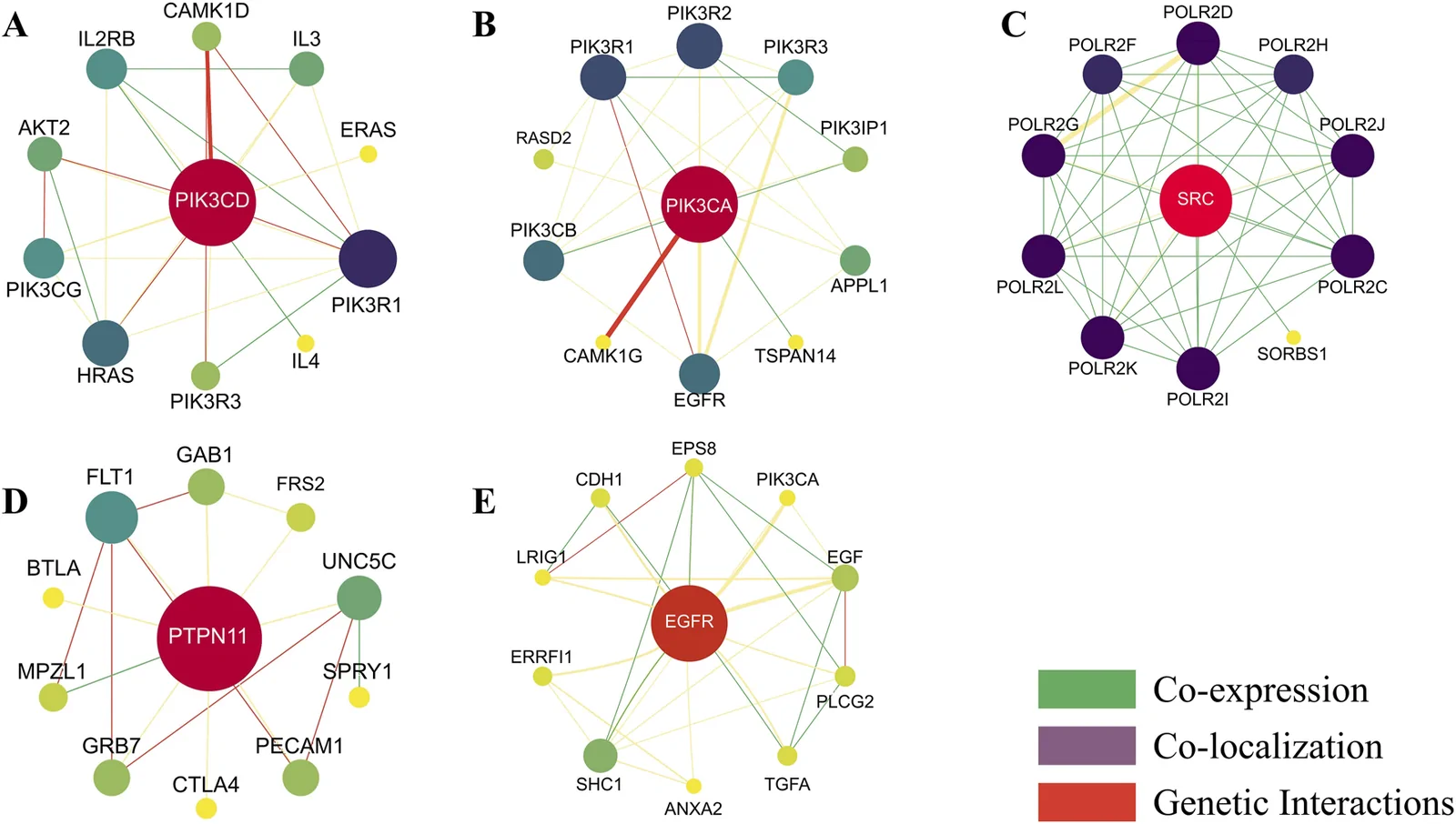
GeneMANIA-based functional association (GMFA) network analysis illustrating genes functionally related to **(A)** PIK3CD, **(B)** PIK3CA, **(C)** SRC, **(D)** PTPN11, and **(E)** EGFR.

**FIGURE 8:**
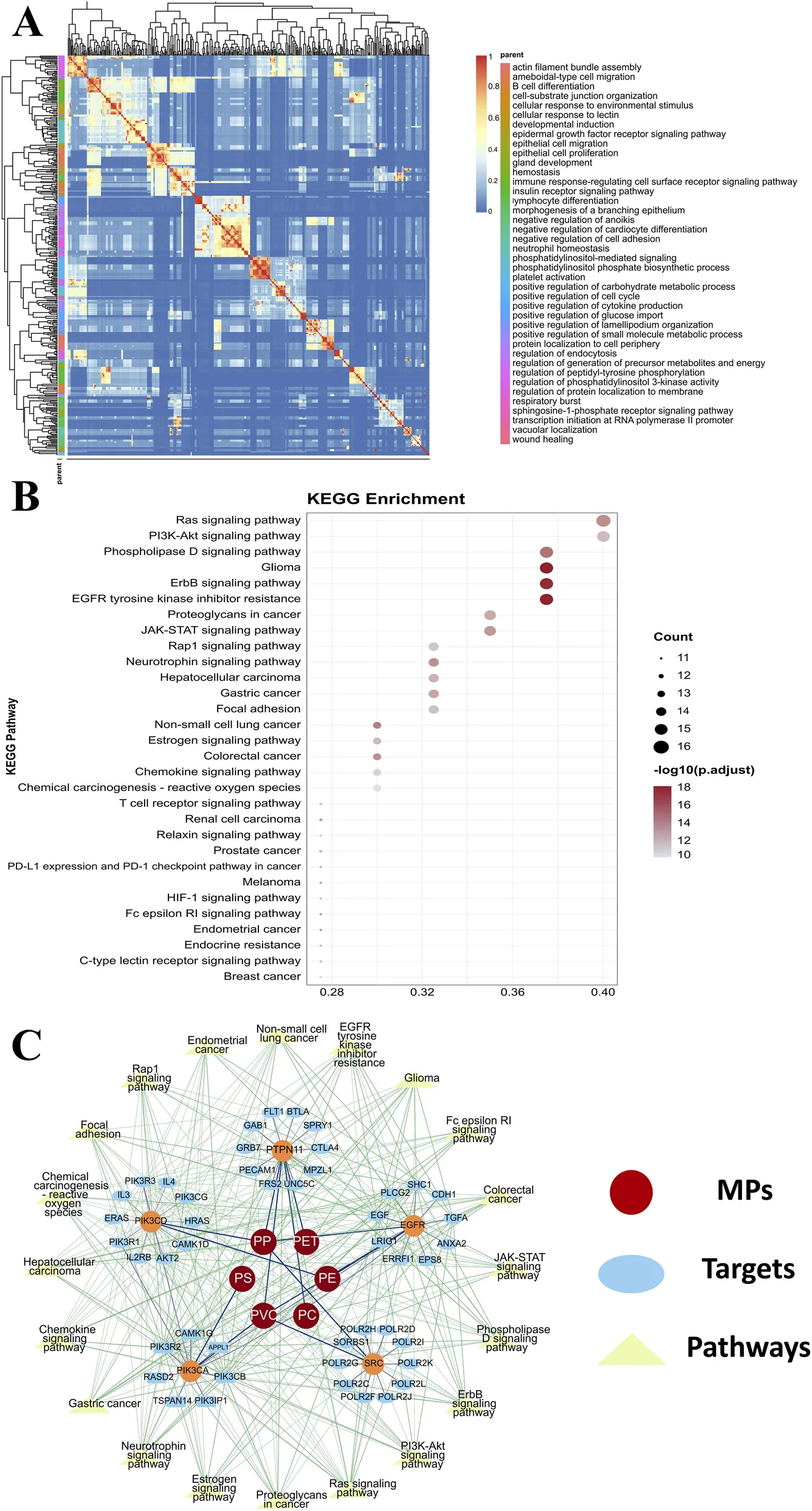
GMFA-ED enrichment and network analysis. **(A)** GMFA-ED GO enrichment analysis. **(B)** GMFA-ED KEGG enrichment analysis. **(C)** Compounds-targets-pathways network. The complete functional gene network of GMFA-ED is provided in Supplementary Figure S10.

### 3.7 Experimental validation of colonic inflammation

To evaluate the effect of MPs on colonic inflammation, disease activity index and colon length were monitored and H&E staining was conducted. The experimental schedule is shown in Figure 9A, and body-weight changes were recorded throughout the study (Figure 9B). From day 11–15, compared with the colitis group, disease activity index of the colitis + MPs group increased (Figure 9C). Compared with normal control group, colon length of the colitis group and the colitis + MPs group both decreased, whereas colon length of the colitis group and the colitis + MPs group remained no significant differences (Figure 9D). Histopathological examination by H&E staining showed that in the healthy control group, the overall architecture of colonic tissue was essentially normal. No obvious loose edema, necrosis, or inflammatory cell infiltration was observed in the mucosal and submucosal layers. In contrast, the other two groups exhibited typical features of colitis, including epithelial damage, crypt distortion, and inflammatory cell infiltration (Figure 9E).

**FIGURE 9:**
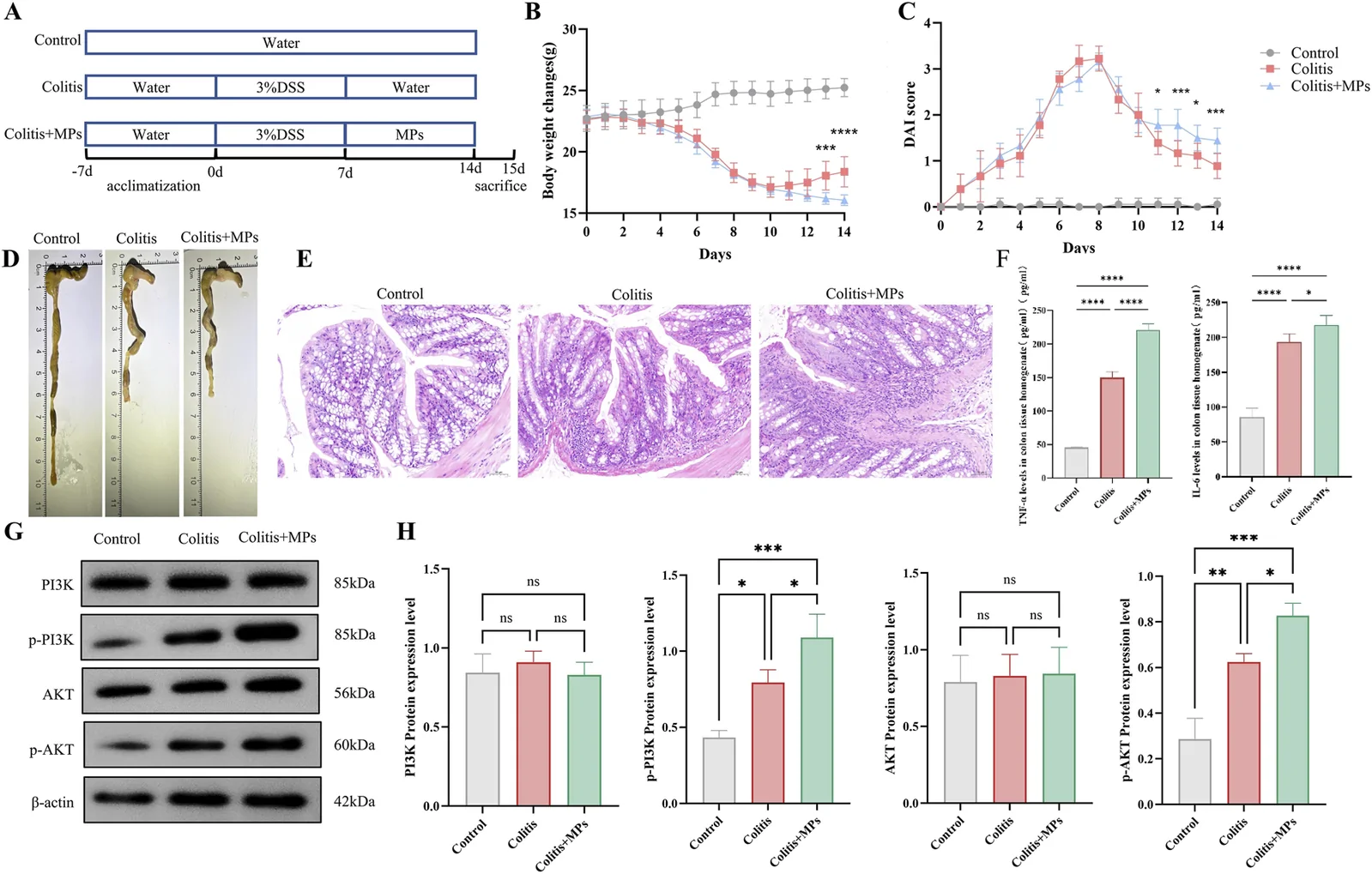
Experimental validation of colitis and PI3K-Akt pathway activation. **(A)** Experimental timeline; **(B)** body-weight changes; **(C)** DAI score; **(D)** representative colon images; **(E)** representative H&E-stained colon sections; **(F)** colonic TNF-alpha and IL-6 concentrations; **(G)** representative western blots of PI3K, p-PI3K, AKT, p-AKT, and beta-actin; and **(H)** densitometric quantification normalized to beta-actin. Data are presented as mean ± SD. ns, not significant; *P < 0.05; **P < 0.01; ***P < 0.001; ****P < 0.0001.

To evaluate PI3K–Akt pathway activation, immunofluorescence staining and western blotting were performed. Representative p-AKT and p-PI3K staining patterns are shown in Figures 1A,C, respectively. At the available magnification, the p-AKT signal appeared more prominent in crypt-associated mucosal regions in the DSS-treated sections (Figure 1A). This regional distribution is morphologically compatible with an epithelial-associated pattern, but individual epithelial cell boundaries cannot be resolved in these images and no epithelial marker was co-stained. Therefore, the observation should be regarded as a morphology-based regional description rather than evidence of definitive epithelial-cell localization. Quantitative immunofluorescence analysis of three biologically independent animals per group showed higher normalized fluorescence densities of p-AKT and p-PI3K in both DSS-treated groups than in the normal control group, with further increases in the colitis + MPs group relative to the colitis group (Figures 1B,D). Additional biological replicates are shown in Supplementary Figure S8, and the underlying measurements are provided in Supplementary Tables S1 and S2. Western blotting likewise showed higher p-PI3K and p-AKT levels in the colitis and colitis + MPs groups, whereas total PI3K and AKT levels remained largely unchanged (Figures 9G,H). Together, these results indicate that MPs exposure is associated with increased PI3K and AKT phosphorylation in the inflamed colon. Western blotting of the five docking-supported targets showed higher PIK3CA, PIK3CD, SRC, PTPN11, and EGFR abundance in both DSS-treated groups than in normal controls and further increases in the colitis + MPs group relative to the DSS-colitis group (Supplementary Figures S5–S7).

ELISA was performed to quantify the protein abundances of IL-6 and TNF-α within colonic tissues. Compared with the normal control group, colonic IL-6 and TNF-α levels were elevated in both DSS-treated groups and were further increased in the DSS-colitis + MPs group relative to the DSS-colitis group (Figure 9F).

## 4 Discussion

Over the past 50 years (1970–2019), the global aquatic environment has accumulated around 0.3 million tons of MPs from personal care products (Sun et al., 2020). Plastic packaging constitutes a significant source of human microplastic exposure, as it can contaminate food. It is estimated that humans release 188 tons of MPs annually through foods (Fadare et al., 2020). Previous research indicates that MPs are present in over 90% of bottled water brands, with bottled water containing 22 times more MPs than tap water and predominantly composed of PET fragment structures (Mason et al., 2018; Schymanski et al., 2018). The most commonly detected MPs in food are PET, PE, PP, PS, PVC, PA and PC (Kadac-Czapska et al., 2024), and the total burden of human exposure to MNPLs has been recently estimated to be 2.93 × 1,010 particles/year (Domenech and Marcos, 2021). The presence of MPs was reported in the colons of healthy adults and IBD patients (Lykkemark et al., 2025). Therefore, it is essential to determine the health impacts of MPs exposure, particularly at the intestinal level. To the best of our knowledge, this is the first study to use network toxicity techniques and molecular docking to examine the molecular effects and interaction networks between MPs and UC. These results demonstrated the potential toxicity of MPs, which can cause UC via various targets and routes. This study provides valuable insights into UC prevention and risk assessment in human health.

In this study, ProTox 3.0 was utilized to forecast the oral toxicity and toxicity endpoints. MPs possess an extensive surface area, pronounced surface hydrophobicity, and a propensity for the adsorption of contaminants, such as microbes, heavy metals, and organic pollutants (Alizadeh et al., 2024). These properties could increase the complexity of MPs pollution and corresponding toxicological assessments. Neurotoxicity, nephrotoxicity and disruption of the blood-brain barrier were the most frequent toxicity endpoints predicted in our research. The diminutive size and distinctive surface activity of MPs facilitate their traversal of the blood-brain barrier, resulting in accumulation within the cerebral cortex, hippocampus, and cerebellum (Kwon et al., 2022), potentially leading to neuroinflammation, deficits in learning and memory, and reduced levels of synaptic proteins (Jeong et al., 2022; Wang et al., 2022). Multiple studies have demonstrated that MPs are detrimental to the kidneys or renal cells by inducing oxidative stress and inflammation, diminishing metabolic activity, disrupting cell-cell connections, and triggering autophagy and endoplasmic reticulum stress (Goodman et al., 2022; Meng et al., 2022; Wang et al., 2021).

In addition to the aforementioned toxicity endpoints, research has revealed that microplastic exposure is substantially correlated with diminished sperm count and motility, underscoring the potential reproductive health hazards associated with microplastic pollution (Zhang et al., 2024). Although possible toxicity in the digestive system was not anticipated, research has indicated that microplastics can traverse the digestive tract after ingestion, and smaller microplastics can infiltrate the intestinal epithelium and enter the circulatory system (Wu et al., 2022), leading to the digestive tract serving as a primary accumulation site and toxic target for the ingested MPs (Zhao et al., 2024). Research indicates that the concentration of MPs is positively correlated with the severity of IBD, as IBD patients exhibit a significantly higher concentration in feces compared to healthy individuals (Yan et al., 2022). A new publication showed that MPs are associated with biomarkers of intestinal inflammation (Lykkemark et al., 2025), suggesting that MP exposure may be linked to the onset and progression of intestinal diseases. MPs are more prone to infiltrate damaged intestinal epithelial cells, instigating the excessive proliferation of intestinal stem cells, which disrupts colonic epithelial homeostasis and facilitates their translocation to various systems (Xie et al., 2023). Absorption, digestion, or inhalation via mucosal contact likely results in the detection of microplastics (MPs) in the human bloodstream in a size-dependent manner (Leslie et al., 2022). The bloodstream subsequently transports them to organs, where they cause intestinal toxicity, metabolic disruption, reproductive toxicity, neurotoxicity, and immunotoxicity through mechanisms such as oxidative stress, apoptosis, and specific pathways (Liu et al., 2023).

A total of 265 potential targets of MPs that cause UC were identified using network toxicology. Through the molecular actions of DNA-binding transcription factors and protein tyrosine kinase activity, enrichment analysis indicates that MPs may accumulate in membrane rafts or microdomains and contribute to the regulation of the PI3K-Akt signaling pathway, MAPK cascade, oxidative stress, and biological process of wound healing (Agrawal et al., 2024). According to clinical studies, exposure to MPs weakens the gut epithelial barrier, induces changes in intestinal flora, interferes with lipid metabolism, results in oxidative stress, and causes the release of inflammatory markers (Salim et al., 2014; Zhu et al., 2018).

Using three different algorithms in the PPI network, seven possible main targets were identified, and molecular docking was performed with the related MPs. Based on the binding energy being less than −5.0 kcal/mol, five targets were finally chosen as the main targets of MPs that cause UC. The five main targets are PIK3CA, PIK3CD, SRC, PTPN11, and EGFR. Based on the criterion of binding energy < −5.0 kcal/mol, five targets were ultimately identified as the hub targets of MPs inducing UC. The five primary targets are PIK3CA, PIK3CD, SRC, PTPN11, and EGFR. PIK3CA and PIK3CD are components of phosphatidylinositol-3-kinase (PI3K). The primary role of PI3K is phosphorylation, which initiates a cascade of intracellular signaling by phosphorylating other proteins. These signals pertain to cellular functions, including cell growth, migration, and survival. The PIK3CA protein is involved in the PI3K-Akt-mTOR pathway, which helps cells survive, can turn on signals that prevent cell death, and plays an important role in the development of UC (Dobyns and Mirzaa, 2019). Activating PIK3CD can lead to overactivity of the PI3K signaling pathway, which may cause abnormal activation and development of CD4^+^ T cells, increase the number of helper T cells, and reduce the formation of regulatory T cells (Bier et al., 2019). SRC encodes a non-receptor tyrosine kinase that regulates tyrosine phosphorylation at many cell surface receptors. Research shows that SRC activity and protein levels are higher in cancerous cells from UC, and the activation of the SRC proto-oncogene is one of the first steps in the development of colon cancer from UC (Cartwright et al., 1994). Additional study on the toxicity of plasticizers indicates that SRC is a critical target in breast cancer (He et al., 2024). The protein expressed by PTPN11 belongs to the family of protein tyrosine phosphatases, commonly referred to as SHP-2. PTPN11 polymorphisms encoding SHP-2 serve as indicators of susceptibility to UC. Mice exhibiting intestinal epithelial-specific SHP-2 loss demonstrated impaired development and swiftly manifested severe colitis, with the inflammatory transcription factors Stat3 and NF-κB being overactivated early in the altered colon epithelium (Coulombe et al., 2013; Narumi et al., 2009). The function of EGFR in UC has been investigated extensively. Aberrant activation of the EGFR signaling pathway is linked to the pathophysiology of UC, especially regarding its role in UC-related carcinogenesis (Dubé et al., 2012). Multiple MPs compounds possess strong carcinogenicity, which may be associated with the EGFR signaling pathway.

The GMFA network analysis method for the five main targets improved the results of network toxicology by combining co-expression, physical interactions, and genetic interactions. Through genetic interactions, genes linked to similar processes have been identified, opening potential treatment avenues. This integrative strategy comprehensively investigated the toxicity targets of MPs in UC patients. The GO enrichment analysis of GMFA-ED focused more on biological processes, molecular functions, and cellular components related to UC than the results from 265 common targets. The KEGG pathways that were enriched, such as the PI3K-Akt signaling pathway, JAK-STAT signaling pathway, T cell receptor signaling pathway, and HIF-1 signaling pathway, also showed how important they are in the toxic effects of MPs. These results enhance the trustworthiness of GMFA analysis and yield a more robust connection between targets through co-expression, physical interactions, and genetic interactions. Ultimately, the compound-target-pathway network offers a comprehensive landscape for a person to understand the influence of MPs in UC. These findings identified important toxicity targets of MPs that cause UC, helping to explain how the toxicity of MPs might lead to disease.

The animal experiments provide preliminary biological support for the computational predictions. Both network toxicology analyses highlight PI3K-Akt pathway as a potentially relevant signaling axis in MPs-associated UC. Consistent with this prediction, our *in vivo* validation showed that MPs exposure was accompanied by increased phosphorylation of PI3K and AKT in colitic colon tissues. The five prioritized proteins were also more abundant in the colitis + MPs group than in the colitis group. Spatially, the p-AKT signal appeared more prominent in crypt-associated mucosal regions at the available magnification (Figure 1A). This morphology-based observation is compatible with, but does not demonstrate, involvement of the epithelial compartment. Because individual epithelial cell boundaries were not resolved and epithelial or other cell-type markers were not co-stained, contributions from epithelial, immune, stromal, and vascular cells cannot be distinguished. Together, these findings suggest that MPs exposure may be associated with enhanced PI3K-Akt pathway activation, as reflected by increased PI3K and AKT phosphorylation, despite the absence of significant changes in total PI3K and AKT abundance. Importantly, these findings should be interpreted as pathway-level validation rather than definitive mechanistic proof. PI3K-Akt signaling is a broadly responsive pathway involved in inflammation, epithelial survival, barrier repair, immune-cell activation, and tissue remodeling. Therefore, the increased phosphorylation of PI3K and AKT observed in the colitis + MPs group may reflect an intensified inflammatory response. This result supports the biological plausibility of our computational prediction.

Although we have adopted various research methods to improve this study, several limitations should be acknowledged. First, because microplastics encompass numerous polymer types, sizes, shapes, surface properties, and additives, this study focused only on the major microplastics detected in the Chinese population; the effects of other microplastic types and exposure characteristics require further investigation. Second, the current animal validation focused on PI3K-Akt pathway activation and the protein expression of the five predicted hub targets. Other predicted signaling pathways, including JAK-STAT, T-cell receptor, and HIF-1 signaling, remain to be experimentally validated. Third, the current validation was performed at the tissue level. At the available magnification, the p-AKT signal could be described only at the regional tissue level on the basis of crypt and mucosal morphology. Individual cell boundaries were not resolved, and no cell-type-specific co-staining was performed. Because PI3K-Akt signaling can be activated in multiple cell types, including epithelial cells, macrophages, fibroblasts, endothelial cells, and lymphocytes, the present data cannot identify the precise cellular compartment responsible for the observed phosphorylation changes. Fourth, only a single high dose (5 mg/kg/d) was set for *in vivo* functional verification, without setting environmentally relevant low-dose gradient groups covering μg–mg/kg range. Fifth, representative intestinal tight junction proteins including Occludin and ZO-1 were not examined in the present study. Subsequent investigations will further characterize intestinal barrier injury induced by microplastic exposure. Sixth, the monomer-based structural models used for computational simulation differ from the intact microplastic particles administered in animal assays, which creates mismatches between our molecular simulation outputs and the real physical properties of microplastics retained in living organisms. Seventh, this study used laboratory purified PS nanoparticles without adsorbed endotoxins or heavy metals for animal verification, which excludes the interference of co-adsorbed environmental pollutants in controlled experimental conditions.

## 5 Conclusion

The network toxicological analysis identifies PIK3CD, PIK3CA, SRC, PTPN11, and EGFR as core toxic hub targets, and clarifies PI3K-Akt, JAK-STAT, T cell receptor and HIF-1 as key mediating pathways linking MPs and UC deterioration. The experimental validation further indicated that MPs exposure was associated with higher colonic abundance of all five prioritized proteins and enhanced PI3K-Akt pathway activation in DSS colitis, supporting the biological plausibility of the computational findings. These results remind us that, although MPs exposure cannot be completely avoided at the population level, reducing the use of plastic products in daily life and dietary habits and developing safer alternatives may be important for UC risk prevention and environmental health protection.

## Data Availability

The datasets presented in this study can be found in online repositories. The names of the repository/repositories and accession number(s) can be found in the article/Supplementary Material.

## References

[B1] Agha R. Mathew G. Rashid R. Kerwan A. Al-Jabir A. Sohrabi C. (2025). Transparency in the reporting of artificial INtelligence – the TITAN guideline. Prem. J. Sci. 10, 100082. 10.70389/PJS.100082

[B2] Agrawal M. Vianello A. Picker M. Simon-Sánchez L. Chen R. Estevinho M. M. (2024). Micro- and nano-plastics, intestinal inflammation, and inflammatory bowel disease: a review of the literature. Sci. Total Environ. 953, 176228. 10.1016/j.scitotenv.2024.176228 39270875 PMC11424240

[B3] Ali N. Katsouli J. Marczylo E. L. Gant T. W. Wright S. Bernardino de la Serna J. (2024). The potential impacts of micro-and-nano plastics on various organ systems in humans. Ebiomedicine 99, 104901. 10.1016/j.ebiom.2023.104901 38061242 PMC10749881

[B4] Alizadeh R. Ghorbani H. Alizadeh A. (2024). Study of adsorption of micro plastics (MPs) using PSF/MIL-100(fe) membrane in 3D printed column from seawater. Int. J. Coast Offshore Environ. Eng(ijcoe) 9, 1–19.

[B5] Bier J. Rao G. Payne K. Brigden H. French E. Pelham S. J. (2019). Activating mutations in PIK3CD and murine CD4+ T cells. J. Allergy Clin. Immunol. 144, 236–253. 10.1016/j.jaci.2019.01.033 PMC6612302 30738173

[B6] Cartwright C. A. Coad C. A. Egbert B. M. (1994). Elevated c-Src tyrosine kinase activity in premalignant epithelia of ulcerative colitis. J. Clin. Invest. 93, 509–515. 10.1172/JCI117000 7509341 PMC293871

[B7] Coulombe G. Leblanc C. Cagnol S. Maloum F. Lemieux É. Perreault N. (2013). Epithelial tyrosine phosphatase SHP-2 protects against intestinal inflammation in mice. Mol. Cell Biol. 33, 2275–2284. 10.1128/MCB.00043-13 23530062 PMC3648065

[B8] Dobyns W. B. Mirzaa G. M. (2019). Megalencephaly syndromes associated with mutations of core components of the PI3KAKT–MTOR pathway: *PIK3CA*, *PIK3R2*, *AKT3*, and *MTOR* . Am. J Med Genet. Pt C 181, 582–590. 10.1002/ajmg.c.31736 31441589

[B9] Domenech J. Marcos R. (2021). Pathways of human exposure to microplastics, and estimation of the total burden. Curr. Opin. Food Sci. 39, 144–151. 10.1016/j.cofs.2021.01.004

[B10] Dubé P. E. Yan F. Punit S. Girish N. McElroy S. J. Washington M. K. (2012). Epidermal growth factor receptor inhibits colitis-associated cancer in mice. J. Clin. Invest. 122, 2780–2792. 10.1172/JCI62888 22772467 PMC3408743

[B11] Estevinho M. M. Midya V. Cohen-Mekelburg S. Allin K. H. Fumery M. Pinho S. S. (2024). Emerging role of environmental pollutants in inflammatory bowel disease risk, outcomes and underlying mechanisms. Gut. 74, 477–486. 10.1136/gutjnl-2024-332523 39179372 PMC11802320

[B12] Fadare O. O. Wan B. Guo L.-H. Zhao L. (2020). Microplastics from consumer plastic food containers: are we consuming it? Chemosphere 253, 126787. 10.1016/j.chemosphere.2020.126787 32464756

[B13] Garcia M. M. Romero A. S. Merkley S. D. Meyer-Hagen J. L. Forbes C. Hayek E. E. (2024). *In Vivo* tissue distribution of polystyrene or mixed polymer microspheres and metabolomic analysis after oral exposure in mice. Environ. Health Perspect. 132, 47005. 10.1289/ehp13435 38598326 PMC11005960

[B14] Geyer R. Jambeck J. R. Law K. L. (2017). Production, use, and fate of all plastics ever made. Sci. Adv. 3, e1700782. 10.1126/sciadv.1700782 28776036 PMC5517107

[B15] Goodman K. E. Hua T. Sang Q.-X. A. (2022). Effects of polystyrene microplastics on human kidney and liver cell morphology, cellular proliferation, and metabolism. ACS Omega 7, 34136–34153. 10.1021/acsomega.2c03453 36188270 PMC9520709

[B16] He N. Zhang J. Liu M. Yin L. (2024). Elucidating the mechanism of plasticizers inducing breast cancer through network toxicology and molecular docking analysis. Ecotoxicol. Environ. Saf. 284, 116866. 10.1016/j.ecoenv.2024.116866 39178760

[B17] Huang W. Song B. Liang J. Niu Q. Zeng G. Shen M. (2021). Microplastics and associated contaminants in the aquatic environment: a review on their ecotoxicological effects, trophic transfer, and potential impacts to human health. J. Hazard. Mater. 405, 124187. 10.1016/j.jhazmat.2020.124187 33153780

[B18] Jeong B. Baek J. Y. Koo J. Park S. Ryu Y. K. Kim K. S. (2022). Maternal exposure to polystyrene nanoplastics causes brain abnormalities in progeny. J. Hazard Mater 426, 127815. 10.1016/j.jhazmat.2021.127815 34823950

[B19] Kadac-Czapska K. Knez E. Grembecka M. (2024). Food and human safety: the impact of microplastics. Crit. Rev. Food Sci. Nutr. 64, 3502–3521. 10.1080/10408398.2022.2132212 36250552

[B20] Kozlov M. (2024). Landmark study links microplastics to serious health problems. Nature. 10.1038/d41586-024-00650-3 38448537

[B21] Kwon W. Kim D. Kim H.-Y. Jeong S. W. Lee S.-G. Kim H.-C. (2022). Microglial phagocytosis of polystyrene microplastics results in immune alteration and apoptosis *in vitro* and *in vivo* . Sci. Total Environ. 807, 150817. 10.1016/j.scitotenv.2021.150817 34627918

[B22] Le Berre C. Honap S. Peyrin-Biroulet L. (2023). Ulcerative colitis. Lancet 402, 571–584. 10.1016/s0140-6736(23)00966-2 37573077

[B23] Leslie H. A. van Velzen M. J. M. Brandsma S. H. Vethaak A. D. Garcia-Vallejo J. J. Lamoree M. H. (2022). Discovery and quantification of plastic particle pollution in human blood. Environ. Int. 163, 107199. 10.1016/j.envint.2022.107199 35367073

[B24] Liu M. Liu J. Xiong F. Xu K. Pu Y. Huang J. (2023). Research advances of microplastics and potential health risks of microplastics on terrestrial higher mammals: a bibliometric analysis and literature review. Environ. Geochem Health 45, 2803–2838. 10.1007/s10653-022-01458-8 36598611 PMC9811881

[B25] Lykkemark J. Picker M. Kim T. Nguyen I. Weinstein K. Chen R. (2025). Microplastics are associated with biomarker of intestinal inflammation in a pilot analysis of the PLANET study. J. Crohn’s Colitis 19, i2242–i2243. 10.1093/ecco-jcc/jjae190.1413

[B26] Mason S. A. Welch V. G. Neratko J. (2018). Synthetic polymer contamination in bottled water. Front. Chem. 6, 407. 10.3389/fchem.2018.00407 30255015 PMC6141690

[B27] Meng X. Zhang J. Wang W. Gonzalez-Gil G. Vrouwenvelder J. S. Li Z. (2022). Effects of nano- and microplastics on kidney: physicochemical properties, bioaccumulation, oxidative stress and immunoreaction. Chemosphere 288, 132631. 10.1016/j.chemosphere.2021.132631 34688716

[B28] Narumi Y. Isomoto H. Shiota M. Sato K. Kondo S. Machida H. (2009). Polymorphisms of PTPN11 coding SHP-2 as biomarkers for ulcerative colitis susceptibility in the Japanese population. J. Clin. Immunol. 29, 303–310. 10.1007/s10875-008-9272-6 19160029

[B29] Olfatifar M. Reza Zali M. Amin Pourhoseingholi M. Balaii H. Ghavami S. B. Ivanchuk M. (2021). The emerging epidemic of inflammatory bowel disease in Asia and Iran by 2035: a modeling study. BMC Gastroenterol. 21, 204. 10.1186/s12876-021-01745-1 33957874 PMC8101120

[B30] Ravindra K. Kaur M. Mor S. (2025). Impacts of microplastics on gut health: current status and future directions. Indian J. Gastroenterol. Off. J. Indian Soc. Gastroenterol. 45, 20–39. 10.1007/s12664-025-01744-0 40268833

[B31] Salim S. Y. Kaplan G. G. Madsen K. L. (2014). Air pollution effects on the gut microbiota: a link between exposure and inflammatory disease. Gut Microbes 5, 215–219. 10.4161/gmic.27251 24637593 PMC4063847

[B32] Schymanski D. Goldbeck C. Humpf H.-U. Fürst P. (2018). Analysis of microplastics in water by micro-Raman spectroscopy: release of plastic particles from different packaging into mineral water. Water Res. 129, 154–162. 10.1016/j.watres.2017.11.011 29145085

[B33] Singh S. Qian A. S. Nguyen N. H. Ho S. K. M. Luo J. Jairath V. (2022). Trends in U.S. health care spending on inflammatory bowel diseases, 1996-2016. Inflamm. Bowel Dis. 28, 364–372. 10.1093/ibd/izab074 33988697 PMC8889287

[B34] Sun Q. Ren S.-Y. Ni H.-G. (2020). Incidence of microplastics in personal care products: an appreciable part of plastic pollution. Sci. Total Environ. 742, 140218. 10.1016/j.scitotenv.2020.140218 32629242

[B35] Wang Y.-L. Lee Y.-H. Hsu Y.-H. Chiu I.-J. Huang C. C.-Y. Huang C.-C. (2021). The kidney-related effects of polystyrene microplastics on human kidney proximal tubular epithelial cells HK-2 and Male C57BL/6 mice. Environ. Health Perspect. 129, 057003. 10.1289/EHP7612 33956507 PMC8101928

[B36] Wang S. Han Q. Wei Z. Wang Y. Xie J. Chen M. (2022). Polystyrene microplastics affect learning and memory in mice by inducing oxidative stress and decreasing the level of acetylcholine. Food Chem. Toxicol. 162, 112904. 10.1016/j.fct.2022.112904 35257813

[B37] Wang R. Li Z. Liu S. Zhang D. (2023). Global, regional and national burden of inflammatory bowel disease in 204 countries and territories from 1990 to 2019: a systematic analysis based on the global burden of disease study 2019. BMJ Open 13, e065186. 10.1136/bmjopen-2022-065186 36977543 PMC10069527

[B38] Wu P. Lin S. Cao G. Wu J. Jin H. Wang C. (2022). Absorption, distribution, metabolism, excretion and toxicity of microplastics in the human body and health implications. J. Hazard. Mater. 437, 129361. 10.1016/j.jhazmat.2022.129361 35749897

[B39] Xie S. Zhang R. Li Z. Liu C. Chen Y. Yu Q. (2023). Microplastics perturb colonic epithelial homeostasis associated with intestinal overproliferation, exacerbating the severity of colitis. Environ. Res. 217, 114861. 10.1016/j.envres.2022.114861 36410465

[B40] Yan Z. Liu Y. Zhang T. Zhang F. Ren H. Zhang Y. (2022). Analysis of microplastics in human feces reveals a correlation between fecal microplastics and inflammatory bowel disease status. Environ. Sci. Technol. 56, 414–421. 10.1021/acs.est.1c03924 34935363

[B41] Zhang C. Zhang G. Sun K. Ren J. Zhou J. Liu X. (2024). Association of mixed exposure to microplastics with sperm dysfunction: a multi-site study in China. EBioMedicine 108, 105369. 10.1016/j.ebiom.2024.105369 39342804 PMC11663775

[B42] Zhao B. Rehati P. Yang Z. Cai Z. Guo C. Li Y. (2024). The potential toxicity of microplastics on human health. Sci. Total Environ. 912, 168946. 10.1016/j.scitotenv.2023.168946 38043812

[B43] Zhu D. Chen Q.-L. An X.-L. Yang X.-R. Christie P. Ke X. (2018). Exposure of soil collembolans to microplastics perturbs their gut microbiota and alters their isotopic composition. Soil Biol. Biochem. 116, 302–310. 10.1016/j.soilbio.2017.10.027

