# Supplementary material for: Microplastic exposure is associated with enhanced PI3K-Akt signaling in DSS-induced colitis: integrated bioinformatics analyses and in vivo validation
Source: Front Pharmacol. 2026 Sep 18;17:1875549. doi: 10.3389/fphar.2026.1875549 (PMC13630634; doi:10.3389/fphar.2026.1875549)
Supplement: Supplementary file 1 [file DataSheet1.pdf]

Contents

This supplement contains ten supplementary figures and two supplementary tables.

Supplementary Figure 1. The morphology of microplastics (TEM)
Supplementary Figure 2. Particle size of microplastics (nano-flow cytometry)
Supplementary Figure 3. Zeta potential of microplastic particles
Supplementary Figure 4. Western blot bands of PI3K, p-PI3K, AKT, p-AKT, and β-actin
Supplementary Figure 5. Western blot bands of PIK3CA, PIK3CD, SRC, PTPN11, EGFR, and β-actin
Supplementary Figure 6. Cropped western blot bands of PIK3CA, PIK3CD, SRC, PTPN11, EGFR
Supplementary Figure 7. Quantitative analysis of PIK3CA, PIK3CD, SRC, PTPN11, EGFR
Supplementary Figure 8. Additional biological replicates of p-AKT and p-PI3K immunofluorescence
Supplementary Figure 9. Degree-based ranking of the PPI network
Supplementary Figure 10. Functional gene network of the GMFA-ED dataset
Supplementary Table 1. Quantitative analysis of p-AKT immunofluorescence intensity
Supplementary Table 2. Quantitative analysis of p-PI3K immunofluorescence intensity

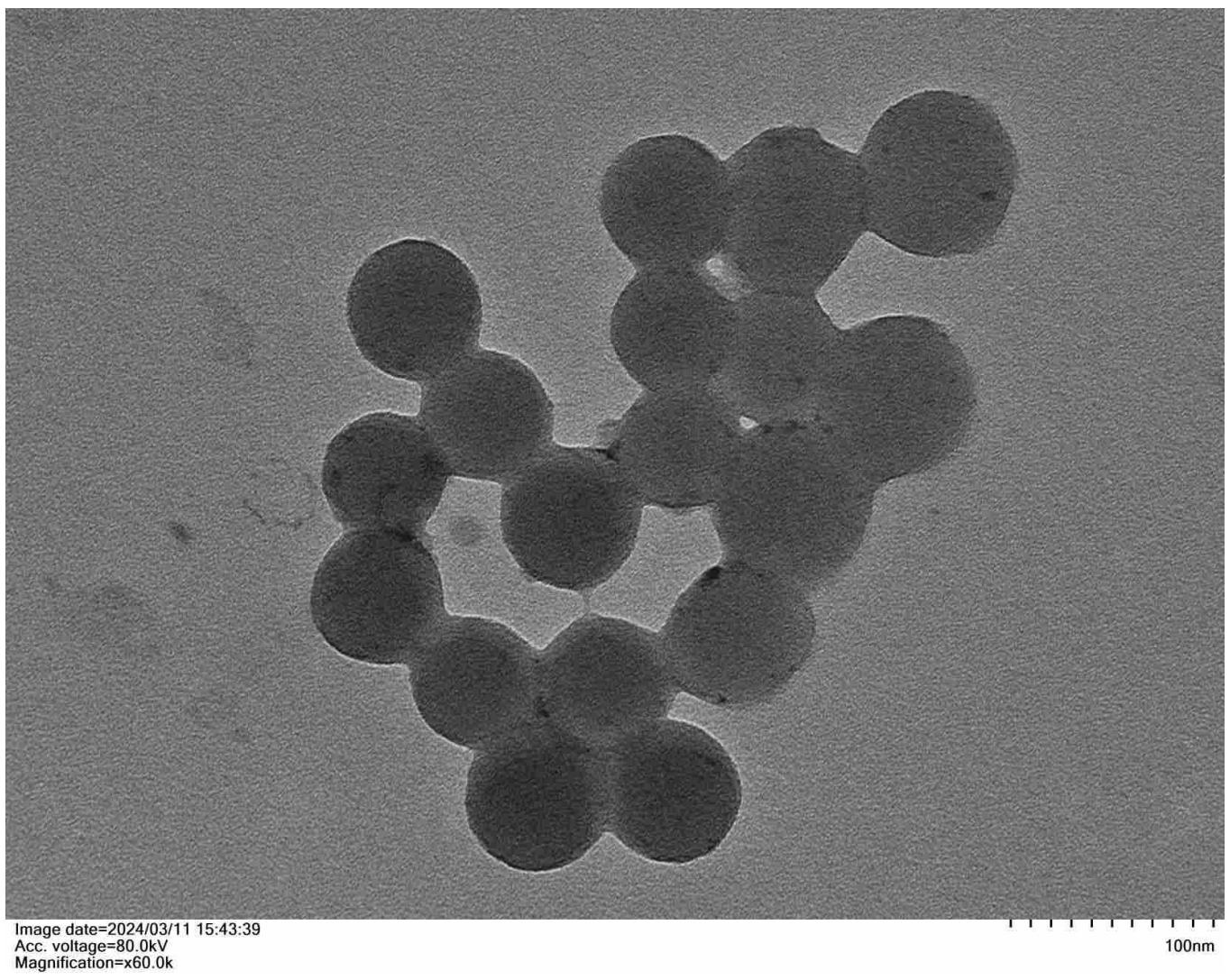

Supplementary Figure 1. The morphology of microplastics was characterized by transmission electron microscopy (TEM). Typical images showed that the microplastics were irregular, approximately spherical particles with clear edges and good dispersion, and the particle size was approximately 100 nm.

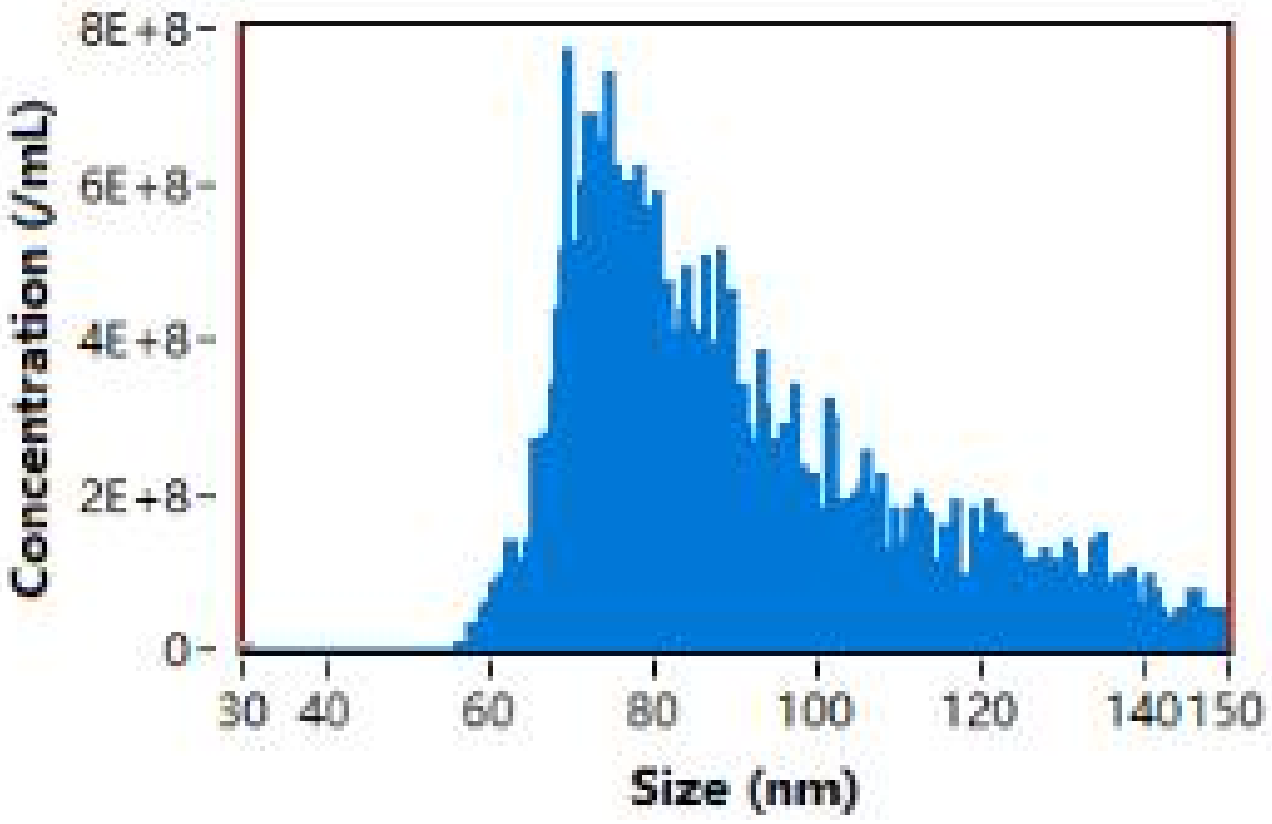

Supplementary Figure 2. The particle size of microplastics was detected by nano-flow cytometry. The results showed that the average particle size of the prepared microplastics was 91 nm, with a relatively uniform particle size distribution and no obvious agglomeration. This result is consistent with the particle morphology characteristics observed by TEM.

System

Temperature (°C):	25.0	Zeta Runs:	12
Count Rate (kcps):	220.4	Measurement Position (mm):	2.00
Cell Description:	Clear disposable zeta cell	Attenuator:	10

Results

			Mean (mV)	Area (%)	St Dev (mV)
Zeta Potential (mV):	-13.9	Peak 1:	-15.8	90.0	6.15
Zeta Deviation (mV):	8.76	Peak 2:	5.58	10.0	5.64
Conductivity (mS/cm):	0.0375	Peak 3:	0.00	0.0	0.00
Result quality	See result quality report				

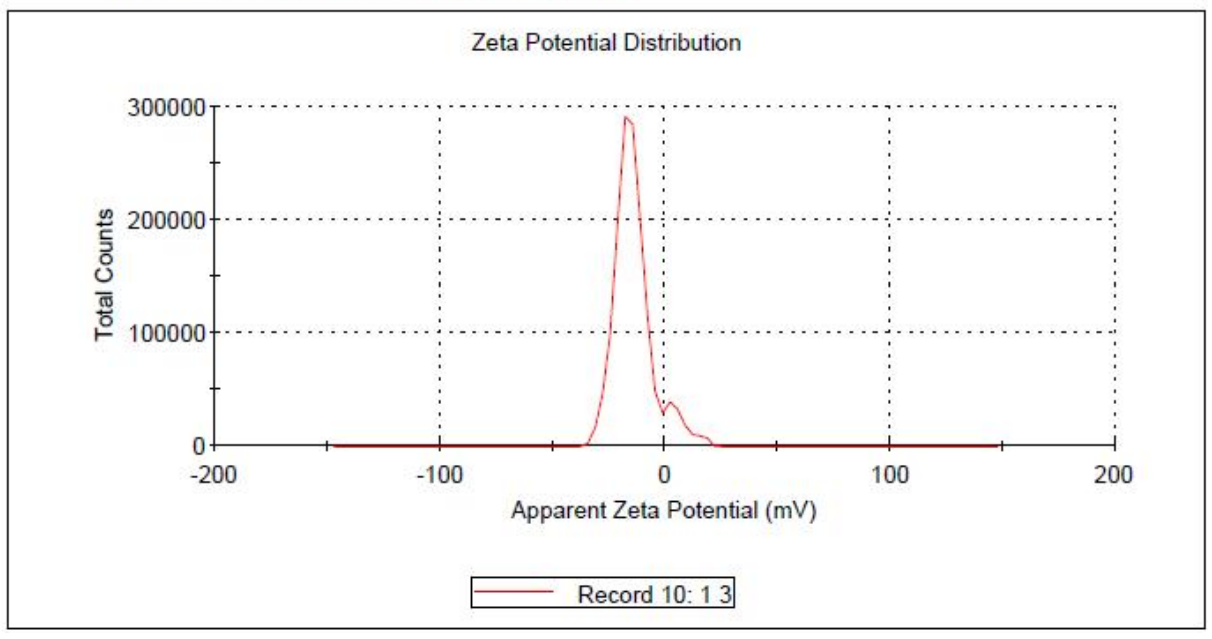

Supplementary Figure 3. The zeta potential of microplastic particles was calculated based on measurements of their electrophoretic migration velocity in an electric field. The results showed that zeta potential was −13.9 mV, indicating a negatively charged particle surface.

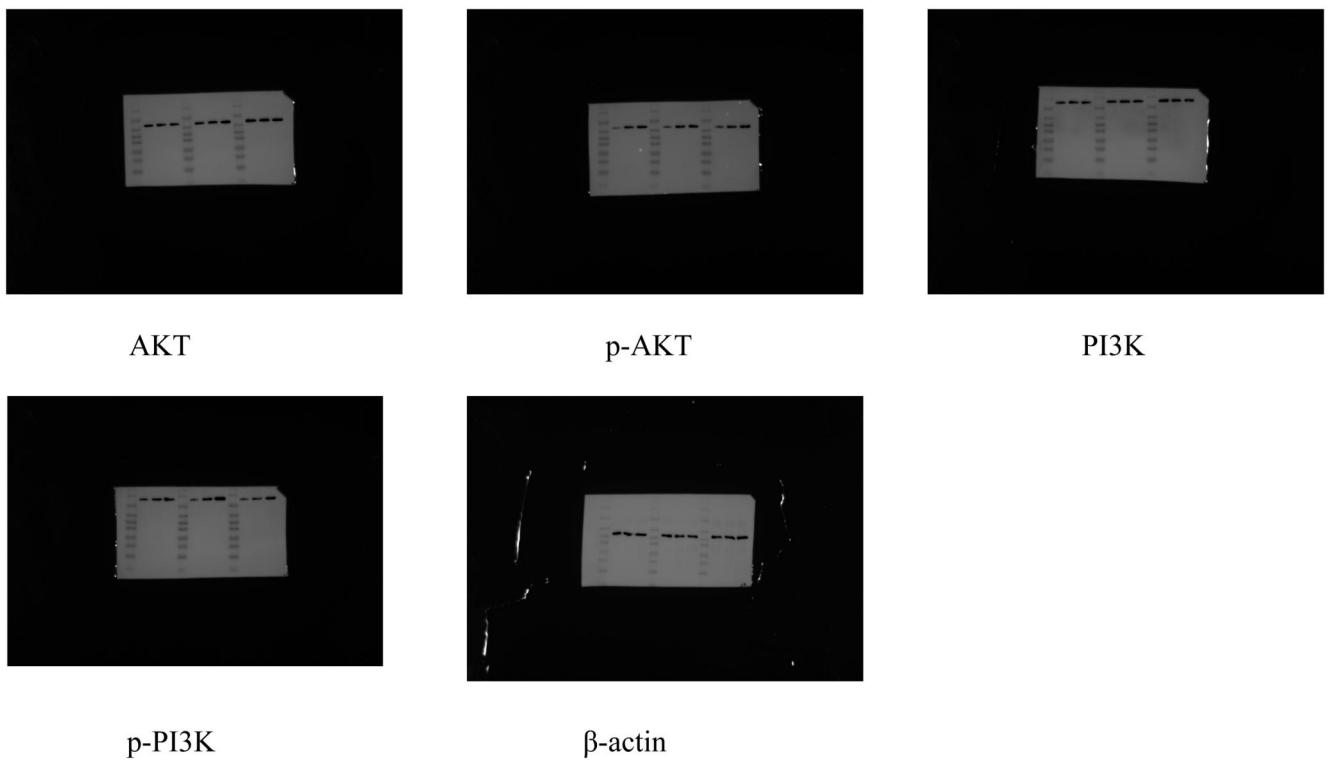

Supplementary Figure 4. Representative western blot bands of PI3K, p-PI3K, AKT, p-AKT, and β-actin in the normal control, DSS-colitis, and DSS-colitis + MPs groups.

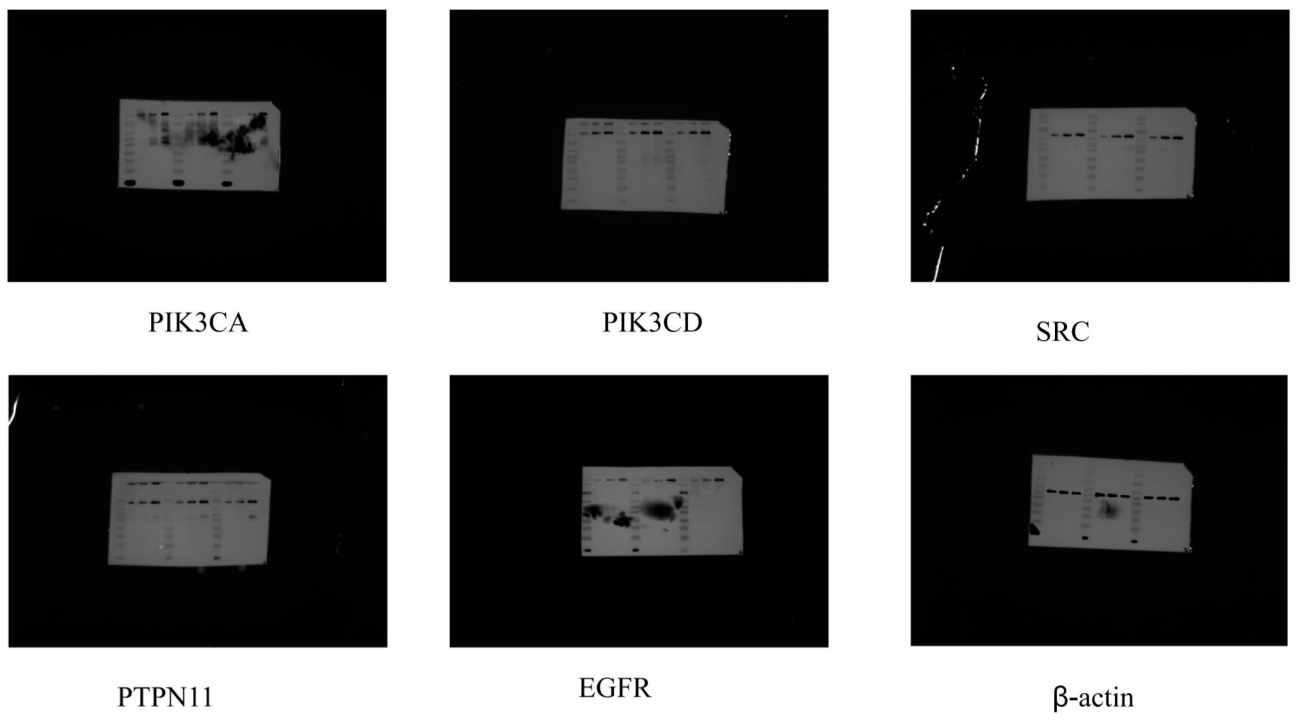

Supplementary Figure 5. Representative western blot bands of PIK3CA, PIK3CD, SRC, PTPN11, EGFR, and β-actin in the normal control, DSS-colitis, and DSS-colitis + MPs groups.

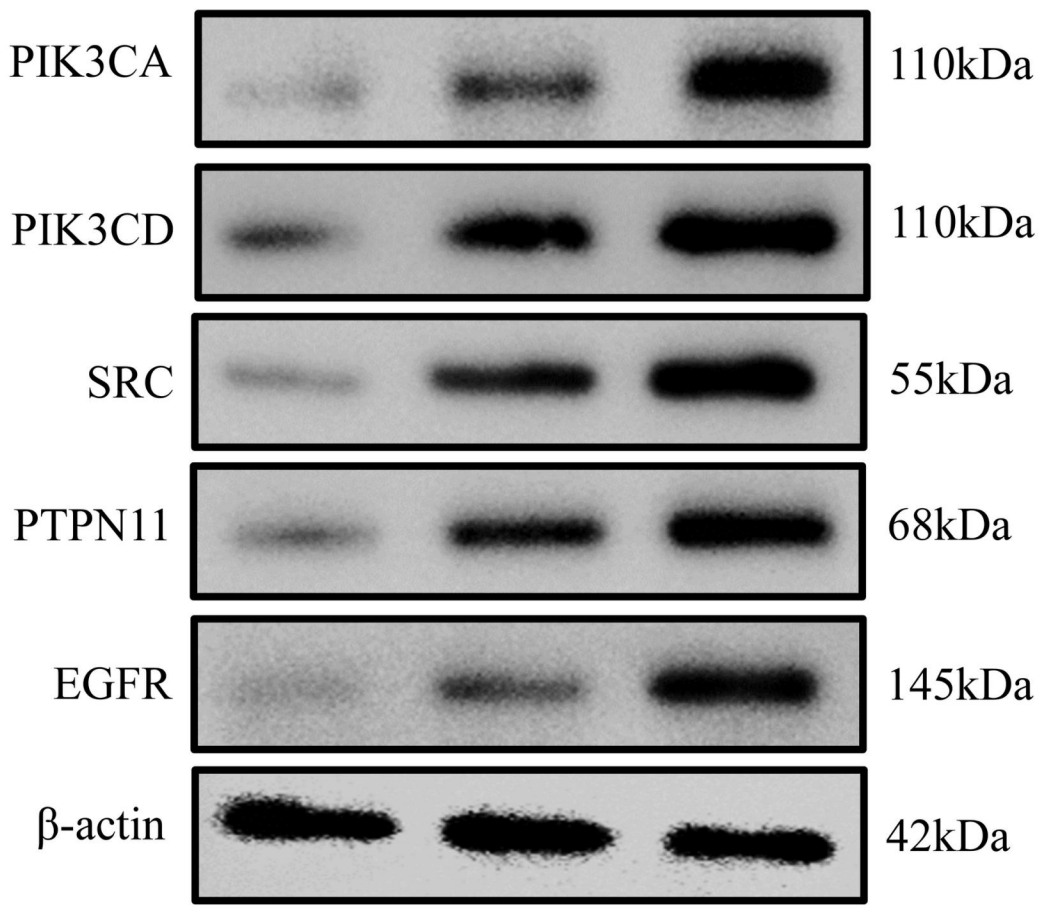

Supplementary Figure 6. Cropped representative western blot bands of PIK3CA, PIK3CD, SRC, PTPN11, EGFR, and β-actin in colonic tissues from the normal control, DSS-colitis, and DSS-colitis + MPs groups. The blots are representative of six biologically independent animals per group.

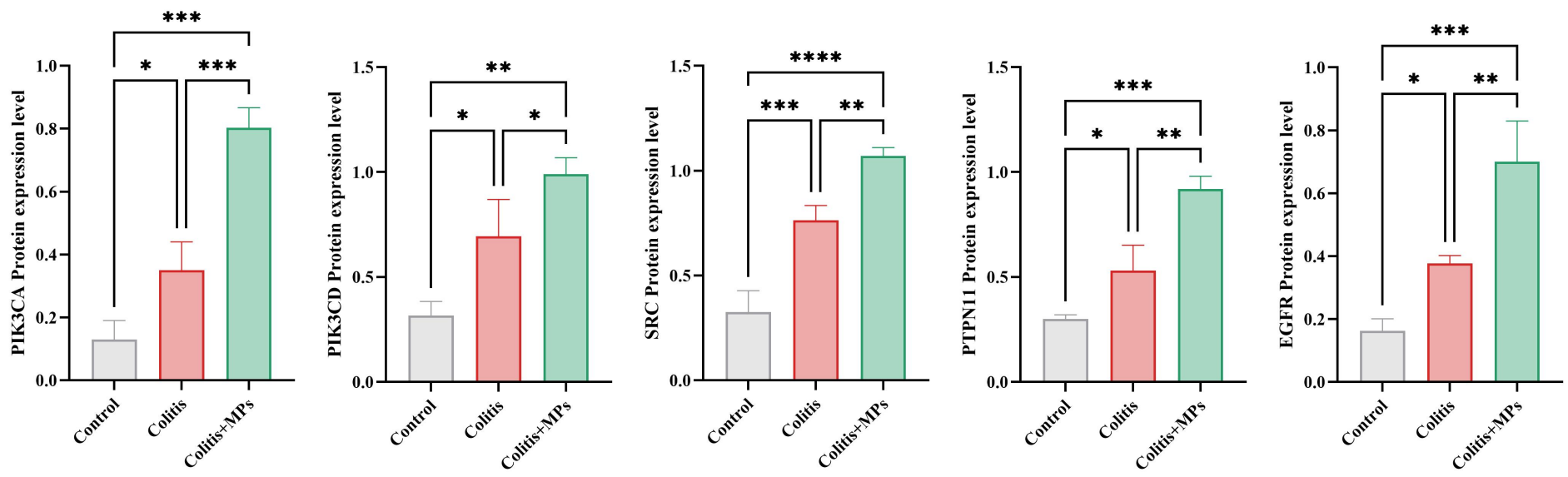

Supplementary Figure 7. Quantitative analysis of PIK3CA, PIK3CD, SRC, PTPN11, and EGFR protein expression normalized to β-actin. Data are presented as mean ± SD from six biologically independent animals per group. For each protein, differences among the three groups were analyzed using one-way ANOVA followed by Tukey's HSD multiple-comparisons test. The symbols indicate multiplicity-adjusted P values: ns, not significant; *P < 0.05; **P < 0.01; ***P < 0.001; and ****P < 0.0001.

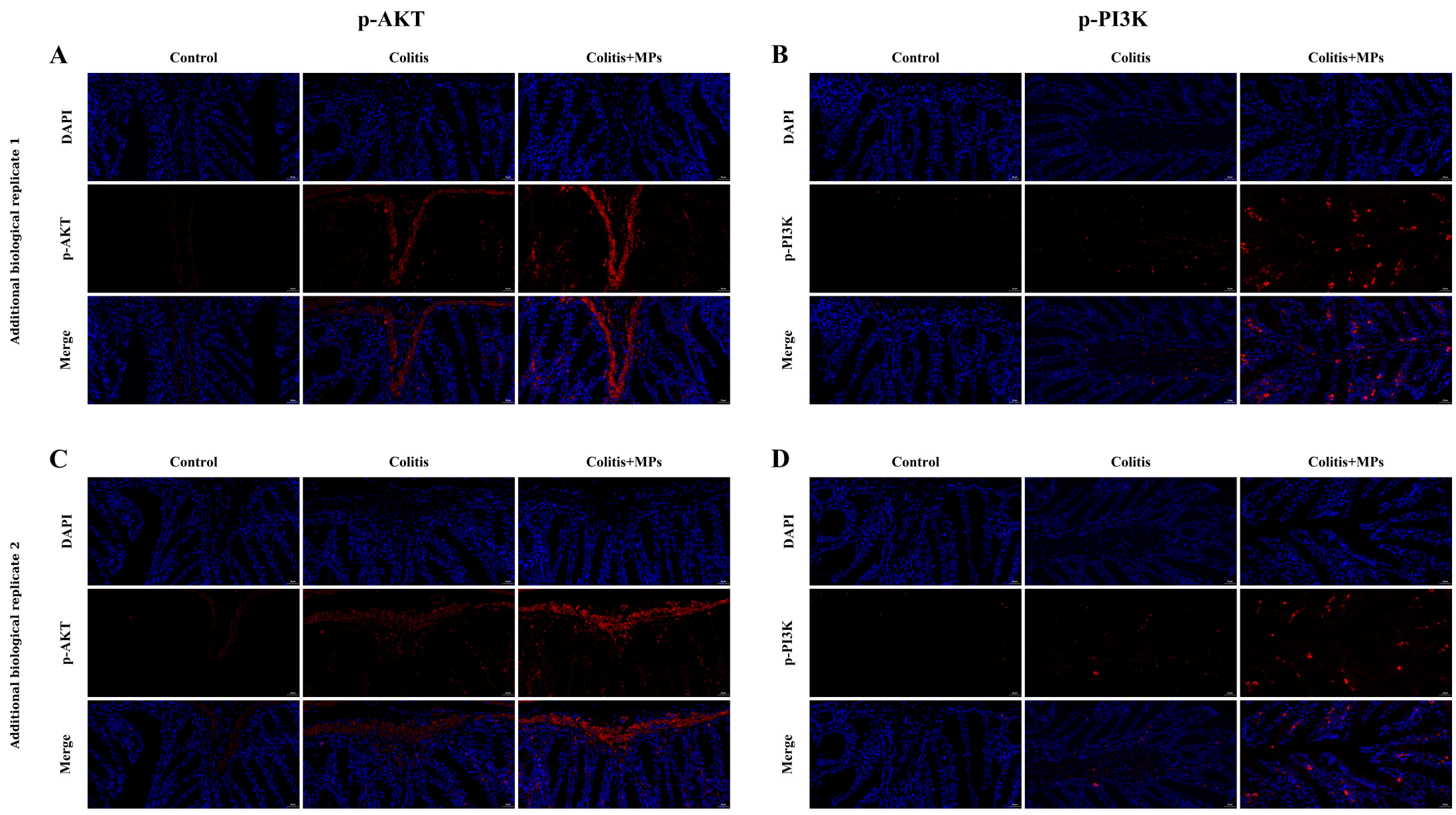

Supplementary Figure 8. Additional representative immunofluorescence images of p-AKT and p-PI3K in colonic tissues. (A, C) p-AKT staining and (B, D) p-PI3K staining in the normal control (NC), colitis, and colitis+MPs groups. Together, panels A–D show two additional biologically independent replicates per marker and group; none of these representative images were used in main-text Figure 9. DAPI is shown in blue, p-AKT or p-PI3K is shown in red, and the corresponding merged images are shown in the bottom rows. Quantification using all three biologically independent samples per group (n = 3) is presented in Figure 9B and 9D. Scale bars = 25 μm. NC, normal control; MPs, microplastics.

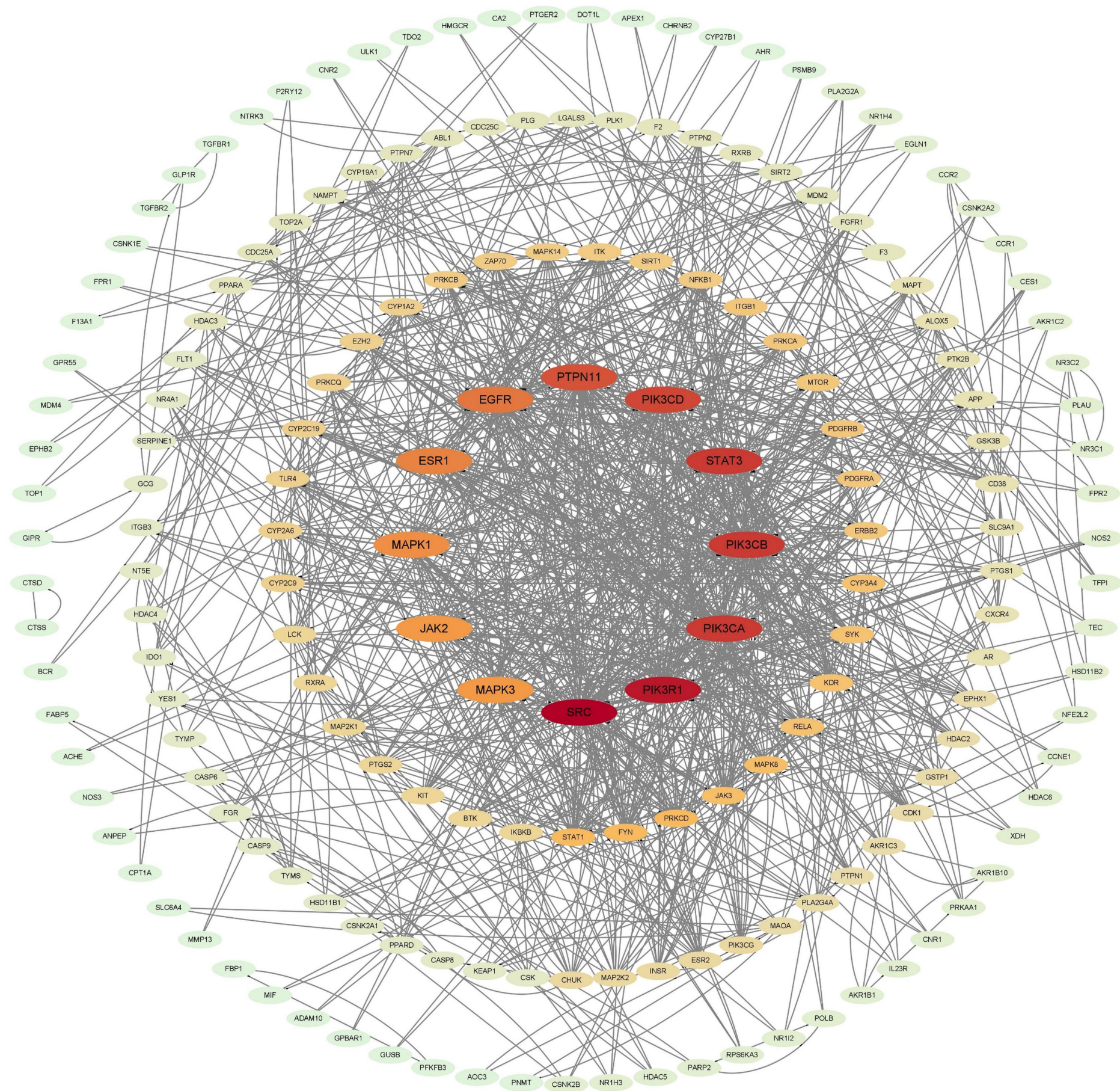

Supplementary Figure 9. Degree-based ranking of the PPI network. Nodes are arranged and colored according to degree centrality, with higher-degree nodes displayed toward the center and in darker colors.

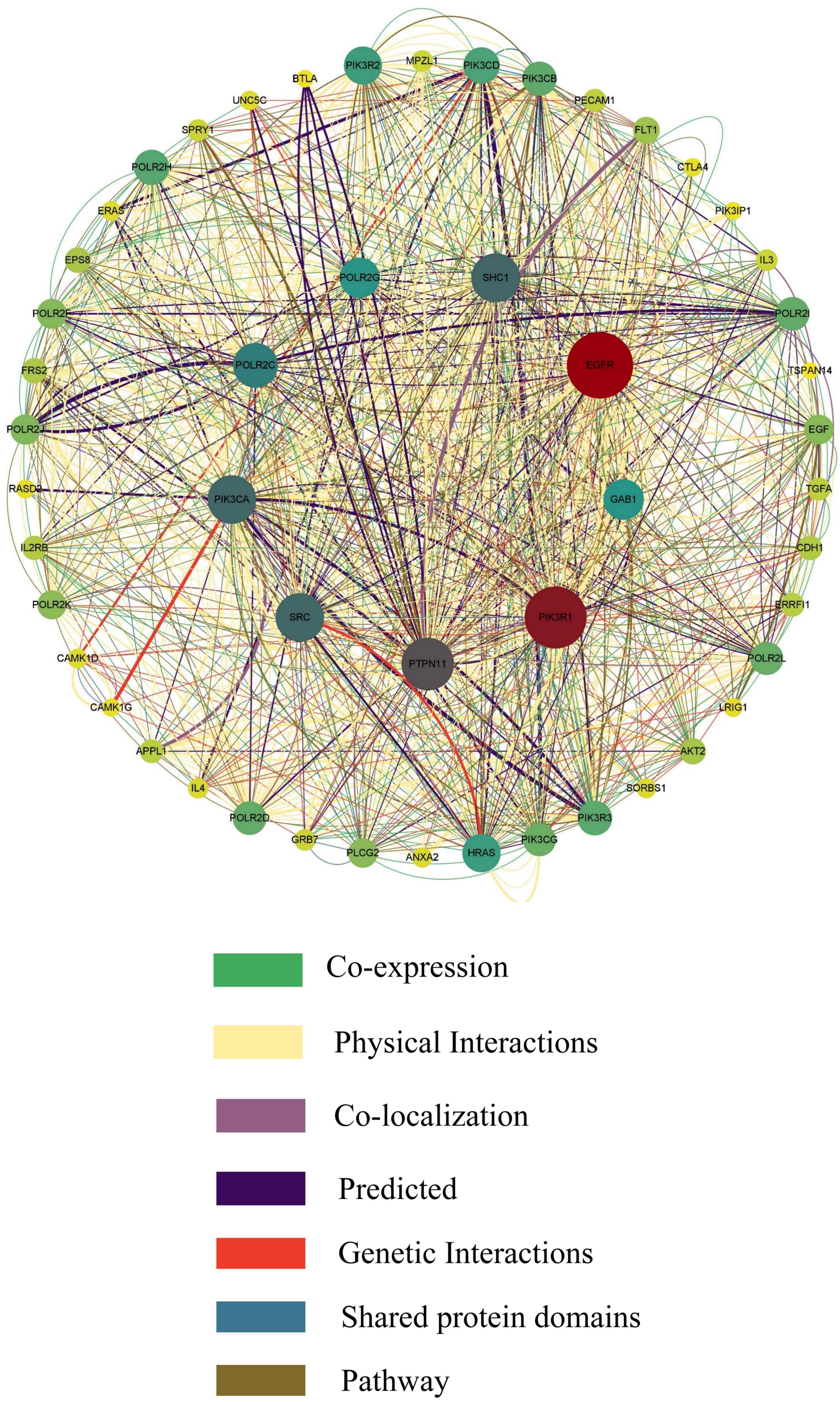

Supplementary Figure 10. Functional gene network of the GMFA-ED dataset. The integrated network comprises 51 unique targets and depicts co-expression, physical interactions, co-localization, predicted interactions, genetic interactions, shared protein domains, and pathway relationships. Edge colors correspond to the interaction categories shown in the legend.

Supplementary Table 1. Quantitative analysis of p-AKT immunofluorescence intensity

Group	Replicate	Positive Area (pixel²)	Integrated Optical Density (IOD)	Measured Area (pixel²)	Normalized Density (IOD / Measured Area)
Normal control	1	10,254	9,964.3568	1,894,053	0.00526
Normal control	2	12,478	10,247.6580	1,894,053	0.00541
Normal control	3	13,245	11,247.6941	1,894,053	0.00594
DSS-colitis	1	20,203	18,660.3975	1,894,053	0.00985
DSS-colitis	2	20,895	16,799.0723	1,894,053	0.00887
DSS-colitis	3	24,915	17,305.8652	1,894,053	0.00914
DSS-colitis + MPs	1	36,934	27,386.6875	1,894,053	0.01446
DSS-colitis + MPs	2	36,169	28,753.0586	1,894,053	0.01518
DSS-colitis + MPs	3	45,999	34,461.6221	1,894,053	0.01819

Notes: Positive Area, Integrated Optical Density (IOD), and Measured Area are in pixel units and were quantified using Image-Pro Plus. Normalized density = IOD / Measured Area of the sample. Statistical analysis (Welch's ANOVA followed by Games-Howell multiple-comparisons tests) is reported in the main text (Figure 9).

Supplementary Table 2. Quantitative analysis of p-PI3K immunofluorescence intensity

Group	Replicate	Positive Area (pixel²)	Integrated Optical Density (IOD)	Measured Area (pixel²)	Normalized Density (IOD / Measured Area)
Normal control	1	5,231	10,245.3654	1,894,053	0.00541
Normal control	2	6,574	9,754.3264	1,894,053	0.00515
Normal control	3	5,963	11,254.3689	1,894,053	0.00594
DSS-colitis	1	13,602	20,713.5952	1,894,053	0.01094
DSS-colitis	2	15,062	16,794.1221	1,894,053	0.00887
DSS-colitis	3	12,118	19,095.7246	1,894,053	0.01008
DSS-colitis + MPs	1	36,706	27,531.5762	1,894,053	0.01454
DSS-colitis + MPs	2	30,847	26,394.0430	1,894,053	0.01394
DSS-colitis + MPs	3	34,593	25,818.9116	1,894,053	0.01363

Notes: Positive Area, Integrated Optical Density (IOD), and Measured Area are in pixel units and were quantified using Image-Pro Plus. Normalized density = IOD / Measured Area of the sample. Statistical analysis (Welch's ANOVA followed by Games-Howell multiple-comparisons tests) is reported in the main text (Figure 9).
